# An Integrated Framework for Classifying Intervention Mechanisms and Measurement Methodologies of Lower-Limb Proprioception in Patients with Stroke: A Systematic Review and Exploratory Meta-Analysis

**DOI:** 10.3390/jcm15197629

**Published:** 2026-10-01

**Authors:** Ming-Yu Tian, Min-Young Lee, Min-Ki Jeon, Myoung-Kwon Kim

**Affiliations:** 1 Department of Physical Therapy, Graduate School, Daegu University, Jillyang, Gyeongsan 712-714, Gyeongbuk, Republic of Korea; mingyutian5@gmail.com (M.-Y.T.); babyseungjun12@hanmail.net (M.-Y.L.); dbsrl3095@naver.com (M.-K.J.); 2 Department of Physical Therapy, College of Rehabilitation Sciences, Daegu University, Jillyang, Gyeongsan 38453, Gyeongbuk, Republic of Korea

**Keywords:** stroke, lower limb, proprioception, systematic review, meta-analysis

## Abstract

**Background/Objectives:** This systematic review and exploratory meta-analysis evaluated interventions targeting lower-limb proprioception after stroke, examined their underlying therapeutic mechanisms and assessment methods, and proposed a mechanism-oriented classification framework for organizing the existing evidence. **Methods:** Randomized parallel-group and randomized crossover studies involving adults with stroke and reporting lower-limb proprioception outcomes were eligible. PubMed, Embase, Scopus, Web of Science Core Collection, Cochrane Library, and MEDLINE via Ovid were searched through 31 March 2026. Risk of bias was assessed using the Cochrane Risk of Bias 2 (RoB 2) tool. Interventions were classified as peripheral, central, or combined peripheral and central mechanisms according to predefined operational criteria. Assessment methods and testing procedures were summarized, and comparable joint position reproduction (JPR) angle-error outcomes were synthesized using random-effects meta-analysis. **Results:** Nineteen studies involving 657 participants were included, of which 10 contributed to the exploratory meta-analysis. Considerable heterogeneity was observed across intervention mechanisms, proprioception measures, and testing procedures. Using a random-effects model, the overall pooled effect showed a statistically significant improvement in proprioception outcomes in favor of the intervention groups (SMD = −0.78, 95% CI −1.30 to −0.25, *p* = 0.004). However, substantial between-study heterogeneity remained (I^2^ = 82%), and the 95% prediction interval crossed the null value, indicating uncertainty in the range of effects that might be observed in future comparable studies. No statistically significant differences were found between the mechanism subgroups. The certainty of evidence for the primary proprioceptive outcome was rated as very low. **Conclusions:** The proposed mechanism-oriented classification provides a structured approach for organizing heterogeneous lower-limb proprioceptive interventions after stroke, but it requires further validation. The quantitative findings should be interpreted as exploratory and hypothesis-generating because of substantial heterogeneity, methodological variability, and very low certainty of evidence. Standardized assessment protocols and higher-quality randomized studies are needed to further evaluate proprioceptive rehabilitation after stroke.

## 1. Introduction

Stroke is frequently accompanied by impaired proprioceptive function [1]. Proprioception encompasses the perception of limb position, movement, and force [2,3]. By updating information about limb configuration and motion, proprioception supports balance, movement accuracy, and adaptation to environmental changes. Proprioception depends on both peripheral sensory input and central nervous system processing. Muscle spindles, Golgi tendon organs, joint receptors, and cutaneous mechanoreceptors detect changes in muscle length, muscle tension, joint position, and movement. Spinal cord and brain integrate these afferent signals with other sensory inputs to guide movement [2,4,5,6,7]. Proprioception is therefore not produced by a single receptor or pathway. It results from the combined function of peripheral receptors, sensory pathways, and several areas of the central nervous system.

Stroke-related disruption of this system may impair judgments of joint position and of the direction and extent of movement. Patients may have difficulty identifying the position of the affected limb, recognizing movement errors, maintaining balance, and adjusting their movements during functional activities. Proprioceptive impairment is associated with poorer motor performance and may occur separately from muscle weakness and other motor impairments [1,8]. Therefore, even when muscle strength improves, reduced proprioceptive feedback may continue to limit coordinated movement, balance, motor learning, and functional recovery [9,10]. In addition, stroke damages the central nervous system. Post-stroke proprioceptive impairment is mainly associated with damage to central sensory pathways and disruptions in sensory processing and sensorimotor integration. Neuroimaging studies of post-stroke proprioception, particularly in the upper limb, have associated persistent impairment with lesions involving multiple cortical and subcortical regions [9]. Neuroimaging studies have also demonstrated altered activation patterns during proprioceptive tasks after stroke [11]. These findings suggest that recovery of proprioception after stroke may depend on changes in brain function and the ability of the nervous system to compensate for damaged sensory processing.

Proprioceptive impairment may occur not only in the paretic limb but also, to a lesser extent, in the non-paretic limb [12]. This suggests that lower-limb proprioceptive impairment after stroke may not be limited to one side of the body and may reflect wider damage to the sensory and motor systems. In a cross-sectional study of chronic stroke, poorer ankle position sense was associated with a higher likelihood of balance impairment [13]. Findings linking ankle proprioception to walking performance have varied across studies. Nevertheless, reduced sensory feedback from the lower-limbs may compromise foot placement, balance corrections, and adaptation of gait to changing task demands. Therefore, lower-limb proprioception should be assessed during stroke rehabilitation and should be considered an important target during standing and walking training.

Despite its clinical importance, proprioception has not been clearly addressed in many stroke rehabilitation studies. Interventions described as proprioceptive training may include sensory stimulation, joint-position exercises, balance training, task-oriented training, or a combination of sensory and motor exercises. However, some studies assess the effects of these interventions only through balance, gait, or general lower-limb function without directly measuring proprioception. Functional gains alone therefore cannot establish whether proprioceptive function has also improved. A similar problem is found in previous systematic reviews and meta-analyses. Some reviews have examined proprioception as part of the broader concept of somatosensation and have combined proprioceptive outcomes with tactile, cutaneous, or other sensory outcomes [14,15]. This approach is useful for examining sensory rehabilitation in general, but it makes it difficult to identify the specific effects of interventions on proprioception.

The methods used to measure proprioception also vary considerably across studies. Common methods include joint position reproduction, joint position matching, detection of passive movement, and movement discrimination tests. These methods differ in whether the movement is active or passive, which joint and target angle are tested, whether vision is blocked, which device is used, how many trials are performed, and how the final error is calculated and reported [16,17]. Therefore, outcomes that are all described as measures of proprioception may actually represent different aspects of proprioceptive function and may not be directly comparable. These differences in intervention content, measurement procedures, and outcome reporting make it difficult to compare findings across studies and limit the reliability of meta-analysis. In addition, classifying interventions only by their treatment names may not clearly explain how they are intended to improve proprioception. Interventions with different names may work in similar ways, whereas interventions with the same general name may involve different types of sensory input or motor practice.

A framework is therefore needed to organize both intervention methods and proprioceptive measurements. Measurement methods should also be organized according to what aspect of proprioception is assessed and how the assessment is performed. This may make it easier to compare studies, interpret treatment effects, and design future research. Accordingly, this systematic review and exploratory meta-analysis aimed to classify interventions for lower-limb proprioception after stroke according to their main intervention mechanisms. It also aimed to summarize the measurement methods, assessment devices, testing procedures, and outcome-reporting methods used in previous studies. Through this approach, the present study sought to clarify the current evidence, identify common methodological problems, and provide practical guidance for future research on lower-limb proprioceptive rehabilitation after stroke.

## 2. Materials and Methods

### 2.1. Study Design

This study was conducted as a systematic review in accordance with the Preferred Reporting Items for Systematic Reviews and Meta-Analyses (PRISMA) guidelines [18] and was registered in the International Prospective Register of Systematic Reviews (PROSPERO; registration number CRD420261405360). The PRISMA 2020 checklist is provided in the Supplementary Materials. A structured approach was adopted to systematically identify, screen, and synthesize existing literature on interventions targeting lower-limb proprioception in patients with stroke.

### 2.2. Search Strategy

A comprehensive literature search was conducted in six electronic databases: PubMed, Embase, Scopus, Web of Science Core Collection, the Cochrane Library, and MEDLINE via Ovid. The initial searches were performed in November 2025. The search strategy was constructed around two major concept groups: stroke and proprioception. Stroke-related terms included “stroke,” “apoplexy,” “CVA,” “cerebral stroke,” “cerebrovascular accident,” “cerebrovascular apoplexy,” “cerebrovascular stroke,” “stroke rehabilitation,” and related variants. Proprioception-related terms included “proprioception,” “labyrinthine sense,” “position sense,” “sense of equilibrium,” “sense of position,” and “vestibular sense.” Terms within each concept group were combined using the Boolean operator OR, and the two concept groups were combined using AND.

The search strategy was adapted to the indexing structure, searchable fields, syntax, and filtering functions of each database. In PubMed, search terms were applied to the Title/Abstract fields, and the Article Type filters “Clinical Trial” and “Randomized Controlled Trial” were applied. In Embase, terms were searched in the title, abstract, and keyword fields (:ti,ab,kw) and combined with the indexed term for randomized controlled trials. In Scopus, terms were searched using TITLE-ABS-KEY, with document type restricted to Article and language restricted to English. In the Web of Science Core Collection, the search was conducted using the Topic field without additional search limits. In the Cochrane Library, terms were searched in the title, abstract, and keyword fields (:ti,ab,kw), with English-language and Trials filters applied through the database interface. In MEDLINE via Ovid, terms were searched using the multipurpose (.mp.) field, with limits applied for humans and randomized controlled trials. Detailed platform-specific search information and complete database-specific search strategies are provided in the Supplementary Materials.

The first search update was conducted on March 31, 2026, in PubMed, Embase, Scopus, and the Web of Science Core Collection to identify newly published or newly indexed records. A subsequent search was conducted in the Cochrane Library and MEDLINE via Ovid on September 14, 2026. For this search, the evidence-coverage cutoff was restricted to March 31, 2026, so that all six databases covered the same final search period. In the Cochrane Library, the date cutoff was applied directly through the database interface. For MEDLINE via Ovid, the search results were exported and records published after 31 March 2026, were removed during post-export date screening.

Although database-specific field restrictions, filters, and limits differed according to the functions available on each platform, the underlying search concepts and Boolean combination of terms remained consistent across all databases. Database-level publication-type filters were used where available; however, final eligibility with respect to randomized controlled trial design was determined during study screening according to the prespecified inclusion criteria. Where equivalent restrictions were not applied at the search stage, the corresponding eligibility criteria were applied consistently during subsequent screening.

### 2.3. Eligibility Criteria

#### 2.3.1. Inclusion Criteria

1. Studies including participants diagnosed with stroke based on clinical criteria or confirmed by imaging methods such as MRI or CT.
2. Studies involving interventions intended to improve proprioception, sensorimotor function, or related proprioceptive rehabilitation functions.
3. Studies reporting quantitative measures of lower-limb proprioception (hip, knee, or ankle). These measures included joint position reproduction (JPR), threshold to detection of passive motion (TTDPM), and active movement extent discrimination assessment (AMEDA).
4. Randomized studies, including both parallel-group randomized controlled trials (RCTs) and randomized crossover trials.

#### 2.3.2. Exclusion Criteria

1. Non-randomized controlled studies, studies that do not involve an intervention (e.g., cross-sectional comparisons, baseline comparisons with healthy controls only).
2. Studies that do not report quantitative measures of proprioception.
3. Studies in which proprioception or sensorimotor function was assessed only as an outcome measure but improving proprioceptive or sensorimotor function was not a therapeutic aim of the intervention.
4. Reviews, case reports, conference abstracts, and other non-original research.
5. Studies published in languages other than English.

### 2.4. Study Screening and Selection Procedures

The study selection process was conducted in two stages. In the first stage, titles and abstracts were screened to exclude studies that clearly did not meet the predefined inclusion criteria. In the second stage, the full texts of potentially eligible studies were retrieved and assessed in detail, and those that fully met the inclusion criteria were included in the final analysis.

One reviewer (M.-Y.T.) independently screened the titles and abstracts and assessed the full-text articles according to the predefined eligibility criteria. Uncertain eligibility decisions were discussed with another researcher (M.-K.K.), and final decisions were reached through discussion.

Studies identified through manual reference screening were subjected to the same inclusion and exclusion criteria as those retrieved from database searches. The study selection process was illustrated using a PRISMA flow diagram.

### 2.5. Data Extraction

A standardized data extraction form was developed prior to data collection. The following information was extracted:

Basic study information (author, study location, publication year, study design, sample size, sex distribution, and participant age). Clinical characteristics (stroke phase). Intervention characteristics (experimental interventions, control interventions, and related intervention information). Intervention dosage and study period (session duration, intervention frequency, total intervention period). Outcome characteristics (proprioception assessment methods, outcome indicators, reporting units). Data extraction was performed independently by one researcher (M.-Y.T.). Any uncertain judgments were discussed with a second researcher (M.-K.K.), and the final judgment was reached by discussion.

### 2.6. Outcome Measures

This study primarily focused on lower-limb proprioception outcomes in patients with stroke, including outcomes related to the hip, knee, and ankle joints. The included studies predominantly used JPR, with a smaller number employing TTDPM or other proprioceptive assessment approaches. These assessments generally reported outcomes as angular repositioning errors, threshold angle for passive motion detection, or discrimination accuracy for different movement amplitudes.

### 2.7. Methods for Intervention Mechanism Classification

Based on established neurophysiological principles of proprioception and sensorimotor learning [2,19], we developed an operational framework to classify intervention mechanisms. Classification focused on the intervention component that differed between the experimental and comparator groups rather than on the entire treatment package. Components delivered similarly to both groups were considered background or co-interventions and were not used to determine the mechanism category.

Interventions were categorized into:

1. Peripheral mechanisms, in which the intervention-specific component primarily acted by directly enhancing or modulating proprioceptive afferent input arising from mechanoreceptors in muscles, joints, tendons, or skin. These interventions were characterized by a direct modification of peripheral sensory input rather than by the subsequent central processing of that input.
2. Central mechanisms, in which the intervention-specific component primarily targeted the processing, integration, weighting, interpretation, or utilization of sensory information within the central nervous system. This category included mechanisms involving sensory reweighting, augmented or altered sensory feedback, error correction, motor planning, sensorimotor integration, or internal motor representation.
3. Combined peripheral and central mechanisms, in which the intervention-specific component incorporated both a direct modification of peripheral proprioceptive input and a central sensory processing or sensorimotor integration component. Interventions were assigned to this category when both mechanisms constituted essential elements of the intervention, and neither could reasonably be regarded as incidental or secondary.

**Figure 1:**
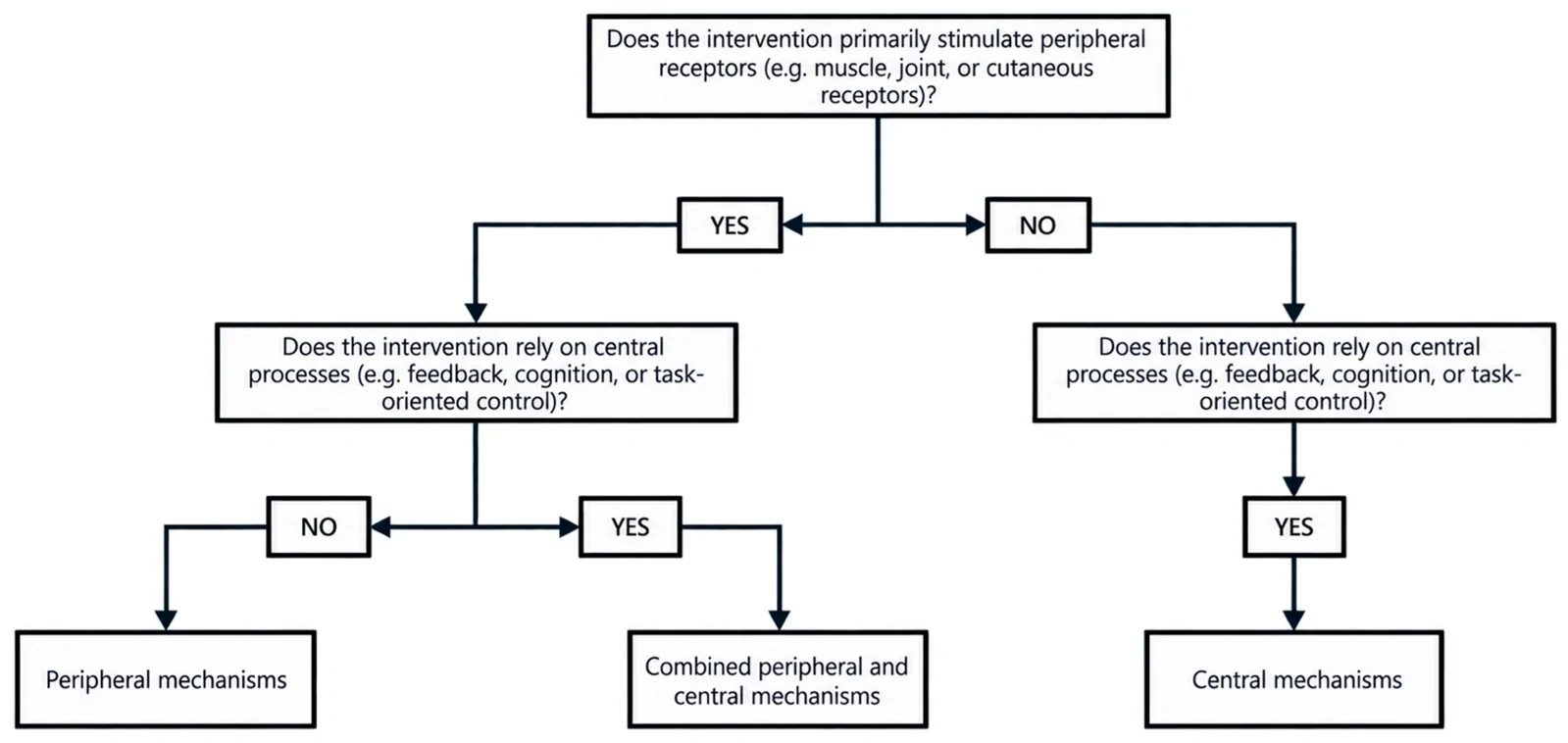
Mechanism classification framework.

This framework was designed to provide a structured and reproducible decision-making process for classifying heterogeneous interventions in proprioception research (Figure 1).

Intervention mechanism classification was performed independently by two reviewers using the predefined classification criteria. Inter-rater agreement before consensus was quantified using Cohen’s κ coefficient. Cohen’s κ was calculated based on the reviewers’ initial independent classifications, prior to any discussion or consensus resolution. Disagreements were subsequently resolved through discussion; when consensus could not be reached, a third reviewer was consulted to determine the final classification. The magnitude of Cohen’s κ was interpreted as follows: <0.00, poor agreement; 0.00–0.20, slight; 0.21–0.40, fair; 0.41–0.60, moderate; 0.61–0.80, substantial; and 0.81–1.00, almost perfect agreement [20].

### 2.8. Methods for Proprioception Assessment and Outcome Reporting Classification

To systematically synthesize proprioception-related outcomes across included studies, a structured data extraction framework was developed based on established proprioception assessment methodologies reported in the literature [16,21]. Specifically, measurement data were extracted and organized into six predefined domains:

1. target joint
2. measurement method and instrument
3. measurement protocol
4. outcome variables and units
5. data processing approach
6. reported statistical format

### 2.9. Risk of Bias and Quality Assessment

#### 2.9.1. Risk of Bias

Risk of bias was independently assessed by two reviewers using the Cochrane Risk of Bias 2 (RoB 2) tool. The assessments focused on the effect of assignments to intervention (the intention-to-treat effect). Each reviewer completed the assessments independently using RoB 2 signaling questions and algorithms. Any disagreements between the two reviewers were resolved through discussion involving a third author [22].

For individually randomized parallel-group trials, the RoB 2 tool for individually randomized parallel-group trials (22 August 2019 version) was used. This tool evaluates risk of bias across five domains:

1. Bias arising from the randomization process

This domain assesses the adequacy of random sequence generation and allocation concealment, as well as baseline comparability between groups.

2. Bias due to deviations from intended interventions

This domain evaluates whether participants adhered to the assigned intervention, and whether interventions, non-compliance, or awareness of group allocation may have influenced the results.

3. Bias due to missing outcome data

This domain considers the extent of missing data, reasons for missingness, and the appropriateness of methods used to handle missing data.

4. Bias in measurement of the outcome

This domain assesses whether outcome measurements were objective and whether outcome assessors were blinded to group allocation.

5. Bias in selection of the reported result

This domain evaluates the presence of selective reporting, including discrepancies between reported outcomes and prespecified protocols.

For the randomized crossover trial, the crossover-specific RoB 2 tool (18 March 2021 version) was used. In addition to the five domains described above, this version includes an additional domain, Domain S: Bias arising from period and carryover effects, which evaluates whether the intervention effect may have differed between periods and whether effects of an intervention administered in one period may have persisted and influenced outcomes in a subsequent period.

For each study, domain-level judgments were categorized as low risk of bias, some concerns, or high risk of bias, according to RoB 2 signaling questions and algorithms.

#### 2.9.2. Certainty of Evidence Assessment

The certainty of evidence was assessed using the GRADE (Grading of Recommendations Assessment, Development and Evaluation) approach [23].

The assessment considered five domains:

1. Risk of bias: Evidence was downgraded when important methodological limitations identified by RoB 2 affected a substantial proportion of the evidence contributing to the pooled estimate and were considered likely to reduce confidence in the estimated effect.
2. Inconsistency: Inconsistency was assessed by considering the direction and magnitude of individual study effects, the overlap of confidence intervals, and statistical heterogeneity, including I^2^. Evidence was downgraded when substantial between-study inconsistency remained unexplained; I^2^ alone was not used as an automatic criterion for downgrading.
3. Indirectness: Evidence was downgraded when important differences were identified between the available evidence and the review question with respect to the population, intervention, comparator, or outcome.
4. Imprecision: Imprecision was assessed based on the 95% confidence interval and the optimal information size (OIS). In the absence of an established minimally important difference for the proprioceptive outcome, the null effect was used as the decision threshold, and an OIS of 800 participants was adopted for continuous outcomes in accordance with GRADE guidance [24]. Evidence was downgraded when the confidence interval crossed the decision threshold or when the available information was insufficient according to the OIS criterion.
5. Publication bias: Evidence was downgraded when publication bias or small-study effects were strongly suspected based on the available evidence, including the pattern of the funnel plot and the corresponding test for funnel plot asymmetry. Funnel plot asymmetry or the statistical test alone was not considered sufficient evidence of publication bias.

The certainty of evidence was categorized as high, moderate, low, or very low.

### 2.10. Data Synthesis and Statistical Analysis

Where studies were sufficiently comparable in terms of intervention characteristics, outcome measures, and study design, quantitative synthesis (meta-analysis) was performed. The prespecified comparator types eligible for quantitative synthesis were general rehabilitation, sham or placebo intervention, or no additional intervention. During the assessment of individual studies, some interventions were found to contain multiple therapeutic components shared by both groups. To operationalize the prespecified comparator criterion in these cases, treatment components delivered equally to both groups were regarded as common co-interventions and were not used to define the intervention contrast. Studies were included in the quantitative synthesis when the remaining comparator condition corresponded to general rehabilitation, sham or placebo, or no additional intervention. If the remaining comparator-specific component represented a proprioceptive intervention classified under a different mechanism, the study was retained in the systematic review but was not pooled in the mechanism-based quantitative synthesis because it represented a comparison between two proprioceptive mechanisms.

For multi-arm studies in which more than one eligible intervention arm shared a common comparator but represented different intervention mechanisms, each eligible intervention-comparator contrast was retained separately to preserve the mechanism-specific classification. To avoid double counting participants in the shared comparator group, the comparator sample size was divided as evenly as possible across the relevant comparisons, while the comparator mean and standard deviation were left unchanged for continuous outcomes.

To be included in the quantitative synthesis, outcome data had to be available in, or derivable into, a form suitable for effect-size calculation, specifically as group means and standard deviations. For studies reporting outcomes at multiple assessment time points, the post-intervention measurement obtained after completion of the full intervention program was used for quantitative synthesis. Outcomes representing only an immediate response to an individual intervention session and follow-up measurements obtained after the post-intervention assessment were not included in the quantitative synthesis.

Although multiple approaches to proprioception assessment were identified in the systematic review, the outcomes for which sufficient data were available for quantitative synthesis were all derived from JPR assessments. Therefore, outcomes obtained using other proprioception assessment approaches were not combined with JPR outcomes. The exploratory meta-analysis was consequently restricted to lower-limb JPR angular repositioning error. For the single study reporting multiple eligible target angles for the same JPR outcome (15°, 30°, and 75°), the intermediate target angle (30°) was selected post hoc as a single representative value to avoid multiple inclusion of the same participants. The selection was based on the position of the target angle within the reported range rather than on the magnitude or statistical significance of the observed effect.

Because the included studies assessed JPR error across different joints and using different measurement and testing procedures, effect sizes were expressed as standardized mean differences (SMDs) with 95% confidence intervals (CIs). For angle-error outcomes, lower values indicated better proprioceptive performance; therefore, negative SMDs favored the experimental group. Meta-analyses were performed using Review Manager 5.4 (RevMan 5.4; The Cochrane Collaboration, Copenhagen, Denmark), with a random-effects model using the DerSimonian–Laird (DL) estimator to account for between-study heterogeneity. Statistical heterogeneity was assessed using τ^2^, Cochran’s Q (χ^2^), and the I^2^ statistic. Mechanism-based subgroup analyses were performed for peripheral mechanisms, central mechanisms, and combined peripheral and central mechanisms, and between-subgroup differences were evaluated using the formal test for subgroup differences.

All supplementary meta-analyses were conducted using R version 4.6.1 with the metafor package. To characterize uncertainty associated with study heterogeneity, 95% prediction intervals were calculated. Sensitivity analyses compared random-effects models using the restricted maximum likelihood (REML) and DL estimators, including their corresponding prediction intervals, and leave-one-out analyses were performed to assess the influence of individual studies on the pooled effect and heterogeneity. Small-study effects were explored using funnel plot inspection and the Pustejovsky–Rodgers regression test for funnel-plot asymmetry when at least 10 independent studies were available. For multi-arm studies contributing more than one eligible comparison, all comparisons from the same study were omitted simultaneously in the leave-one-out analysis. For funnel plot and Pustejovsky–Rodgers analyses, multiple eligible intervention arms from the same study were combined so that each study contributed a single independent effect estimate.

## 3. Results

### 3.1. Study Selection

Searches of six electronic databases yielded 3560 records. After 2038 duplicates or records already identified in earlier searches were removed, 1522 records underwent title and abstract screening. Of these, 1476 were excluded. Retrieval was attempted for 46 reports, of which 10 were unavailable; consequently, 36 reports proceeded to full-text eligibility assessment.

Of the 36 reports assessed for eligibility, 19 were excluded. Three were excluded because they were not published in English, seven because they were not randomized controlled trials, and seven because they did not report an eligible lower-limb proprioception outcome. One report was excluded because the study population included both individuals with stroke and individuals with Parkinson’s disease. Another study was excluded because, although lower-limb proprioception outcomes were reported, the primary therapeutic aim of the intervention was not directed toward improving proprioceptive function. Consequently, 17 studies identified through database searching met the eligibility criteria.

**Figure 2:**
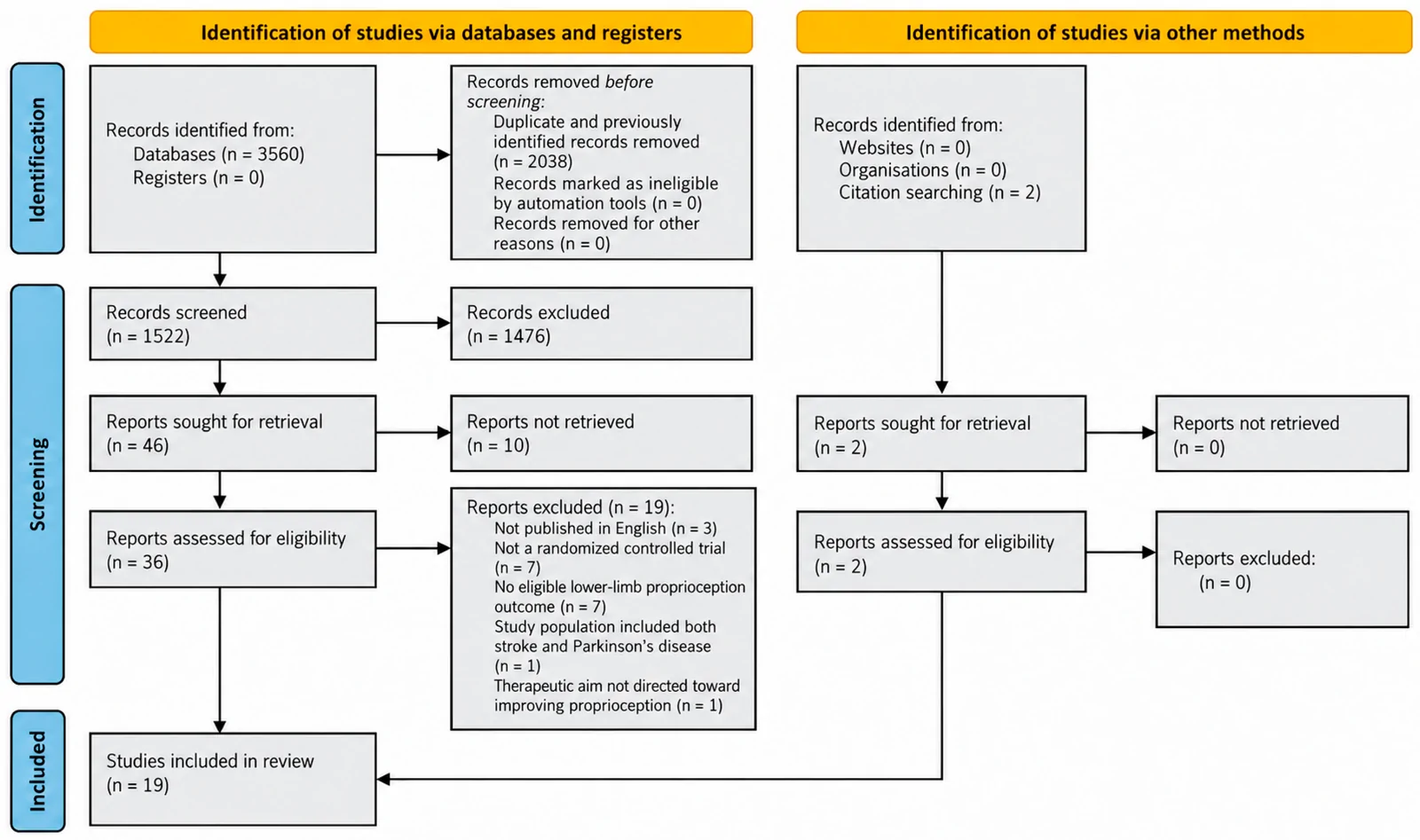
Flowchart of study selection.

In addition, two eligible studies were identified and included through screening the reference list of a systematic review and meta-analysis on lower-limb somatosensory rehabilitation after stroke (Figure 2).

Finally, 19 studies were included in the systematic review [25,26,27,28,29,30,31,32,33,34,35,36,37,38,39,40,41,42,43], and 10 studies contributing 11 intervention-comparator contrasts were included in the meta-analysis [26,27,28,29,30,32,38,40,41,43].

Reasons for exclusion from the meta-analysis included the following: one study [25] reported inconsistent proprioception outcome data; two studies [34,39] evaluated only the immediate effects of a single intervention session rather than outcomes following completion of a multi-session intervention program; two studies [36,37] reported proprioception outcomes in formats other than angular error measures; one study [35] reported only interquartile ranges without sufficient data for effect size calculation; one study [31] presented proprioception outcomes only in graphical form without extractable numerical data. Two studies were excluded because the control intervention had a different proprioceptive intervention than the experimental intervention, so an additional proprioceptive effect in the control group could not be ruled out [33,42].

### 3.2. Study Characteristics

A total of 19 studies were included in this review, comprising randomized controlled trials and randomized crossover trials, with a total of 657 participants with stroke. Among the included studies, 17 investigated individuals with chronic stroke, while 2 studies focused on subacute stroke.

Lower-limb proprioception outcomes primarily included knee proprioception in 10 studies and ankle proprioception in 9 studies. The included studies were conducted in several countries, including China, Korea, Egypt, Turkey, and the United Kingdom.

Intervention duration varied substantially across studies, ranging from single-session immediate interventions to intervention programs lasting up to nine weeks. Considerable variability was also observed in both intervention approaches and proprioception assessment methods across studies (Table 1).

### 3.3. Risk of Bias in Studies

The risk of bias of the included studies was assessed using the Cochrane Risk of Bias 2 (RoB 2) tool. Overall, nine studies were judged to be at low risk of bias and ten were judged to have some concerns; no study was judged to be at high overall risk of bias.

**Figure 3:**
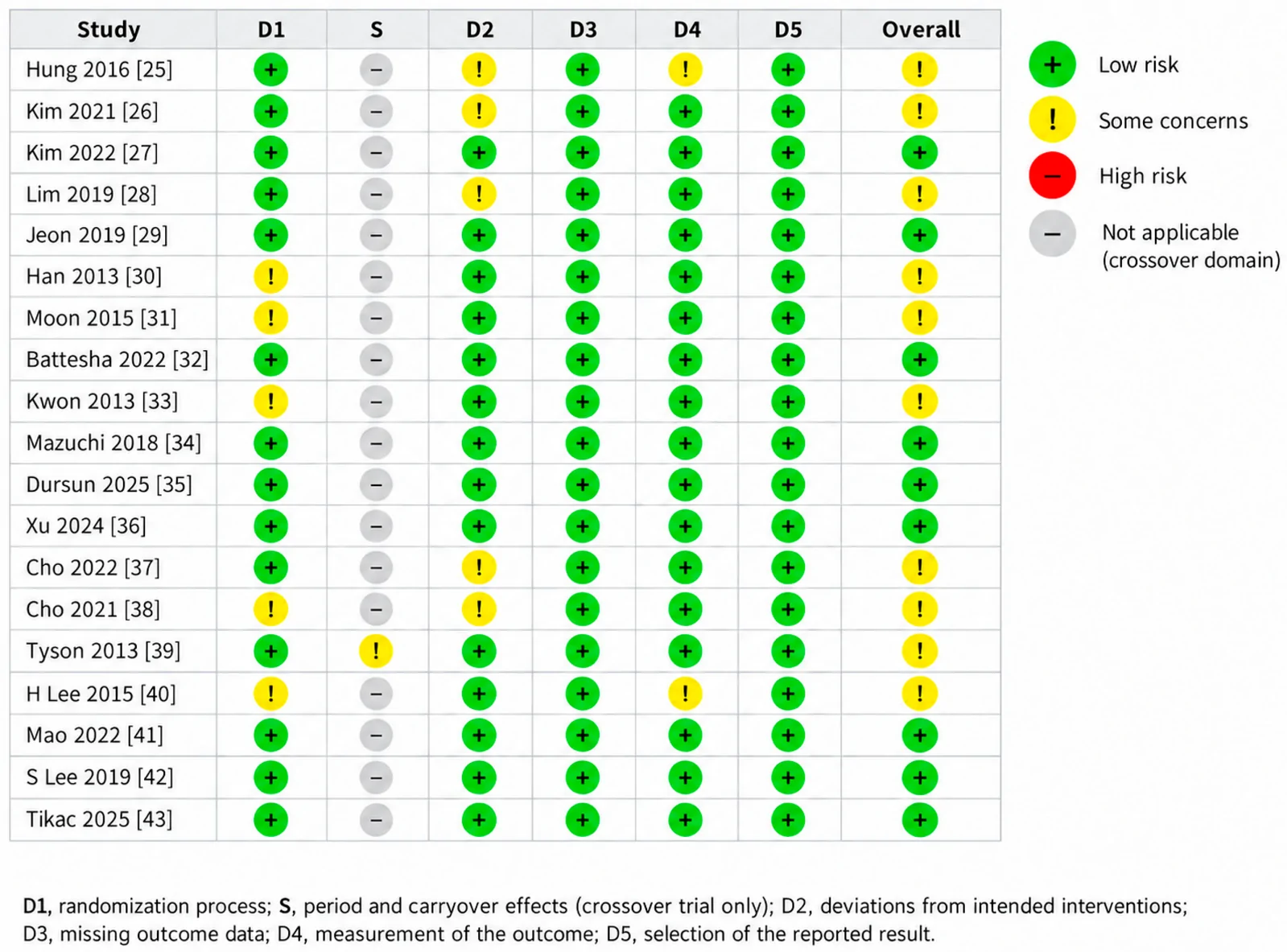
Risk of bias of individual studies [25,26,27,28,29,30,31,32,33,34,35,36,37,38,39,40,41,42,43].

Eighteen parallel-group RCTs were assessed using the RoB 2 tool for individually randomized parallel-group trials. Tyson et al. (2013) [39], which used a randomized crossover design, was assessed separately using the crossover-specific RoB 2 tool. In addition to the five standard domains, this assessment included Domain S, which addresses bias arising from period and carryover effects. Tyson et al. (2013) [39] was judged as having some concerns in Domain S because no washout period between intervention conditions was clearly reported and the available information was insufficient to rule out potential carryover effects. The remaining domains were judged to be at low risk, resulting in an overall judgment of some concerns for this study (Figure 3).

**Figure 4:**
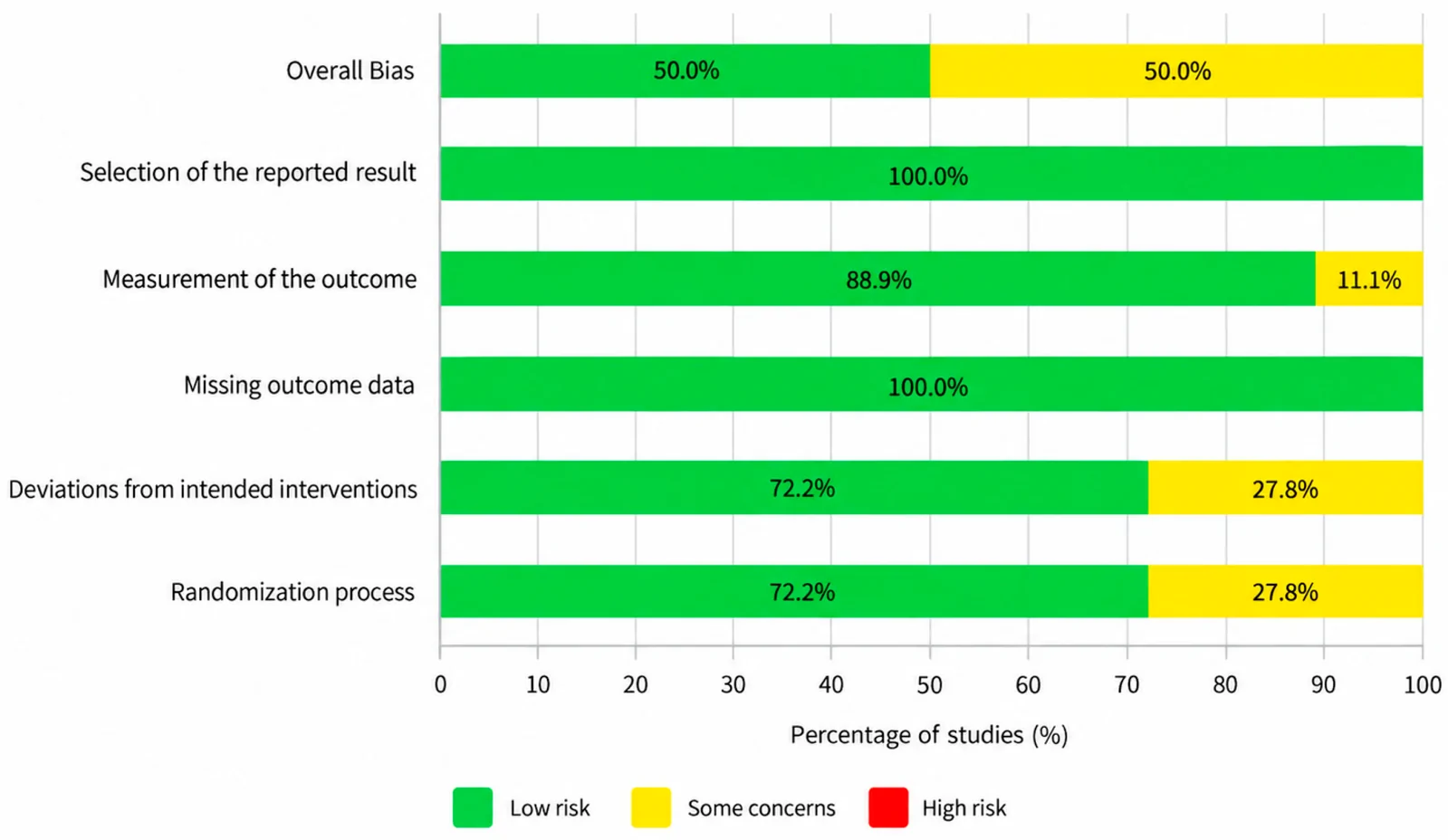
Summary of risk-of-bias judgments across the 18 parallel-group randomized controlled trials.

Among the 18 parallel-group trials, five studies were judged to have some concerns in the randomization process domain (D1), mainly because random sequence generation or allocation concealment was insufficiently described. Five studies were judged to have some concerns in the domain of deviations from intended interventions (D2), primarily because reporting was insufficient to determine whether deviations from the intended interventions occurred or whether such deviations could have influenced the outcomes. All 18 parallel-group trials were judged to be at low risk in the missing outcome data domain (D3). In the measurement of the outcome domain (D4), two studies were judged to have some concerns because outcome assessment involved a degree of assessor judgment and blinding of outcome assessors, or other procedures intended to minimize potential measurement bias, was insufficiently reported. Although there was no direct evidence that outcome measurement was influenced by knowledge of the intervention assignment, potential measurement bias could not be fully excluded. All 18 parallel-group trials were judged to be at low risk in the selection of the reported result domain (D5). The percentage summary shown in Figure 4 is therefore based on the 18 parallel-group trials only; Tyson et al. (2013) [39] was assessed separately using the crossover-specific RoB 2 tool and was not included in this percentage summary (Figure 4).

### 3.4. Classification of Intervention Mechanisms

The 19 included studies contributed 21 intervention arms, which served as classification units and were categorized according to the predefined mechanism-oriented framework (Table 2).

The classification was based on the primary therapeutic component introduced in the experimental group rather than the intervention label alone. In studies where similar rehabilitation components were provided in both experimental and control groups, only the additional mechanism-specific component unique to the experimental intervention was considered for classification. Therefore, the classification reflected the dominant therapeutic mechanism of the intervention rather than an isolated or exclusive mechanism.

Agreement between the two independent reviewers was 95.2% (20/21 classification units), with a Cohen’s κ of 0.921, indicating almost perfect agreement.

Studies classified as central mechanisms mainly included interventions emphasizing visual feedback-based sensorimotor regulation and movement-related feedback control. Typical interventions included Tetrax biofeedback balance training, maze control training, visual feedback ankle strategy exercise, repeated active movement, eye-closed treadmill training, task-specific training, and ankle muscle control training using visual feedback. These interventions primarily aimed to enhance movement correction and sensorimotor control through visually mediated feedback processing. In addition, motor imagery training was also classified as a central mechanism because it emphasized internally mediated motor representation and cognitive motor control processes.

Studies classified as peripheral mechanisms mainly included interventions involving lower-limb sensory training, unstable surface training, underwater proprioceptive training, repeated passive movement, vibration stimulation, transcutaneous electrical nerve stimulation, kinesio taping, and passive joint movement training. These interventions primarily emphasized increased reliance on somatosensory and proprioceptive sensory input during movement or postural control tasks.

Three studies were classified as combined peripheral and central mechanisms because the intervention protocols simultaneously incorporated visual or cognitive sensorimotor regulation together with peripheral sensory stimulation as essential therapeutic components. In these studies, both mechanisms contributed substantially to the intervention design, and the dominant mechanism could not be clearly distinguished within the experimental intervention.

In addition, the intervention and control groups in most studies received the same general rehabilitation treatment, and a few studies did not combine general rehabilitation treatment. However, two studies did not report whether the intervention was the only intervention during the trial period.

### 3.5. Classification of Measurement Methods

The included studies primarily assessed lower-limb proprioceptive function using joint position reproduction (JPR) tests, with active JPR being the most commonly applied assessment method. Several studies additionally employed passive JPR, whereas one study used threshold to detection of passive motion (TTDPM).

Regarding assessment tools, isokinetic dynamometer systems (e.g., Biodex and HUMAC NORM) and electrogoniometers were the most commonly used devices. Several studies additionally incorporated balance training systems or acrylic assessment boards during testing. Outcome measures were primarily reported as angle error (°), although several studies additionally reported success rate (%), absolute error (AE), variable error (VE), or minimum motion perception angle (Table 3).

Mean ± standard deviation was the most common format for data presentation; however, several studies reported outcomes using medians, interquartile ranges, or graphical presentations. Studies differed substantially in their methodological characteristics, including the joints evaluated, measurement methods, outcome metrics, and data-processing procedures.

Several additional sources of methodological heterogeneity could not be fully summarized in Table 3. First, target joint angles and movement directions were inconsistently specified. Second, testing conditions varied across studies, including visual or auditory occlusion, participant position, and whether testing was performed in sitting or standing. Third, the number of repetitions and methods used to calculate summary scores differed substantially; some studies averaged repeated trials at a single target angle, whereas others averaged measurements obtained across multiple target angles. In several studies, these procedures were insufficiently reported.

### 3.6. Subgroup Meta-Analysis Based on Intervention Mechanisms

**Figure 5:**
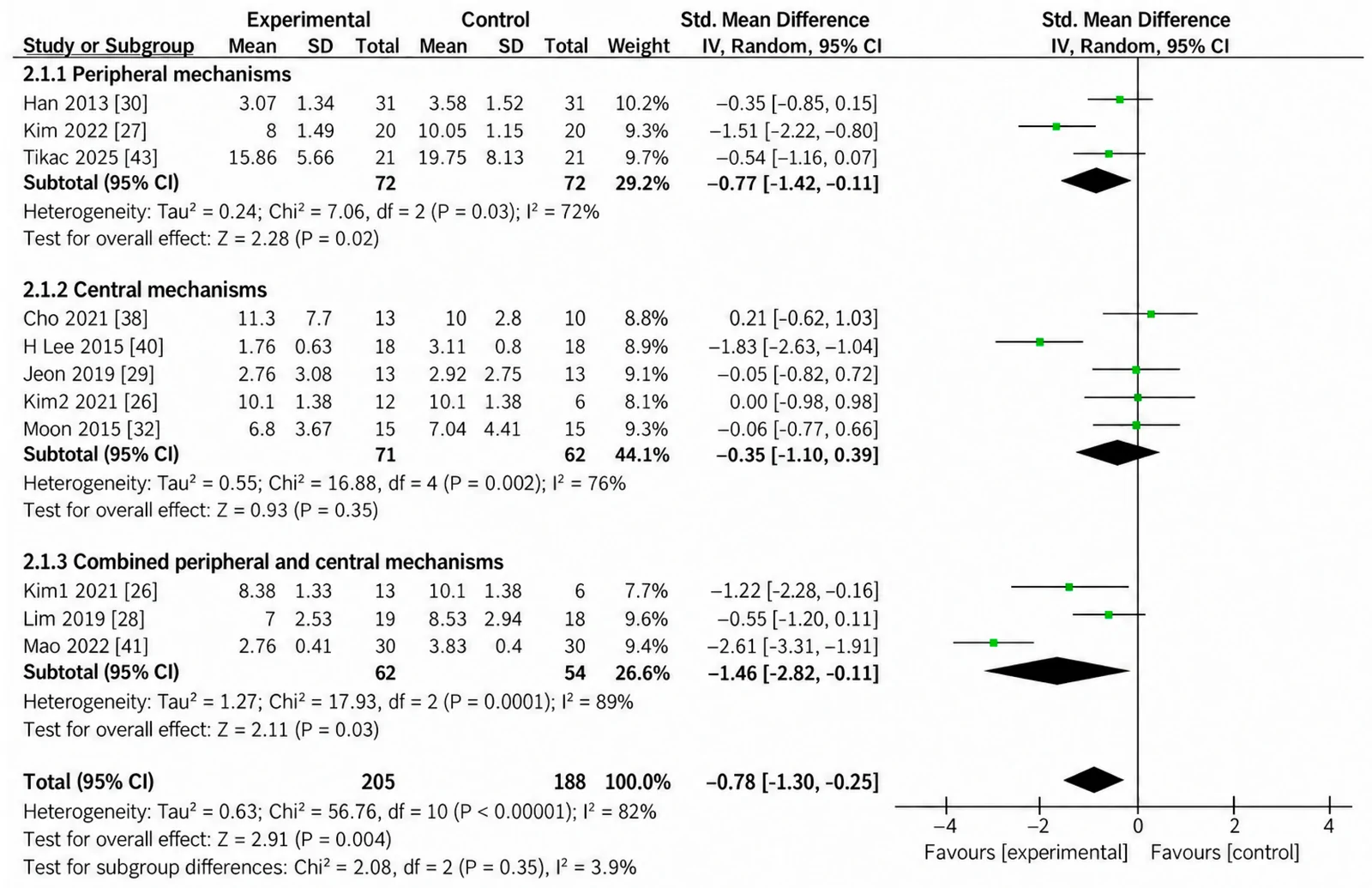
Forest plot of subgroup analysis by intervention mechanism. Green squares indicate individual study effects, with square size proportional to study weight, and horizontal lines represent 95% confidence intervals; the diamond represents the pooled effect estimate [26,27,28,29,30,32,38,40,41,43].

Ten studies were included in the exploratory meta-analysis, contributing 11 effect estimates, and were classified into three subgroups according to the proposed intervention mechanism: peripheral mechanisms, central mechanisms, and combined peripheral and central mechanisms. Using a random-effects model with the DerSimonian–Laird (DL) estimator, the overall pooled estimate favored the experimental groups (SMD = −0.78, 95% CI −1.30 to −0.25, *p* = 0.004), corresponding to lower joint position reproduction error. However, substantial between-study heterogeneity was observed (τ^2^ = 0.63; χ^2^ = 56.76, df = 10, *p* < 0.00001; I^2^ = 82%). The corresponding 95% prediction interval ranged from −2.42 to 0.87 and crossed the null value, indicating considerable uncertainty in the range of true effects that might be observed across comparable study settings (Figure 5).

Three effect estimates were included in the peripheral mechanism subgroup, yielding a pooled SMD of −0.77 (95% CI −1.42 to −0.11, *p* = 0.02), with substantial heterogeneity (τ^2^ = 0.24; χ^2^ = 7.06, df = 2, *p* = 0.03; I^2^ = 72%). Five effect estimates were included in the central mechanism subgroup, for which the pooled estimate was SMD = −0.35 (95% CI −1.10 to 0.39, *p* = 0.35), with substantial heterogeneity (τ^2^ = 0.55; χ^2^ = 16.88, df = 4, *p* = 0.002; I^2^ = 76%). Three effect estimates were included in the combined peripheral and central mechanism subgroup, yielding a pooled SMD of −1.46 (95% CI −2.82 to −0.11, *p* = 0.03), with considerable heterogeneity (τ^2^ = 1.27; χ^2^ = 17.93, df = 2, *p* = 0.0001; I^2^ = 89%). Importantly, the formal test for subgroup differences was not statistically significant (χ^2^ = 2.08, df = 2, *p* = 0.35; I^2^ = 3.9%). Therefore, although the 95% confidence intervals excluded the null value in the peripheral and combined peripheral and central mechanism subgroups, the available evidence did not demonstrate statistically significant differences in treatment effects among the three mechanistic categories (Figure 5).

To assess the sensitivity of the pooled result to the method used to estimate between-study variance, the DL model was compared with a restricted maximum likelihood (REML) model. The DL model yielded an SMD of −0.78 (95% CI −1.30 to −0.25, *p* = 0.004), with τ^2^ = 0.63, I^2^ = 82.38%, and a 95% prediction interval of −2.42 to 0.87. The REML model produced the same pooled point estimate (SMD = −0.78, 95% CI −1.31 to −0.24, *p* = 0.004), with τ^2^ = 0.66, I^2^ = 83.06%, and a 95% prediction interval of −2.46 to 0.91. The close similarity of the pooled estimates, confidence intervals, heterogeneity estimates, and prediction intervals under the two estimators indicated that the overall result was not materially dependent on the choice of between-study variance estimators. Nevertheless, substantial heterogeneity persisted under both models (Table 4).

A leave-one-out sensitivity analysis was subsequently performed to examine whether the overall pooled effect was disproportionately influenced by any individual study. After sequential omission of each study, the pooled SMDs ranged from −0.87 to −0.58, and all corresponding 95% confidence intervals remained below zero. The largest absolute pooled estimate was observed after excluding Cho et al. (2021) [38] (SMD = −0.87, 95% CI −1.41 to −0.33, *p* = 0.002), whereas the smallest absolute pooled estimate was observed after excluding Mao et al. (2022) [41] (SMD = −0.58, 95% CI −0.99 to −0.18, *p* = 0.005). Thus, omission of any individual study did not alter the direction or statistical significance of the overall pooled effect. The largest reduction in heterogeneity occurred after exclusion of Mao et al. (2022) [41], when I^2^ decreased to 66.71% and τ^2^ to 0.27. Nevertheless, substantial residual heterogeneity remained, indicating that the overall between-study inconsistency could not be attributed to a single study (Table 5).

**Figure 6:**
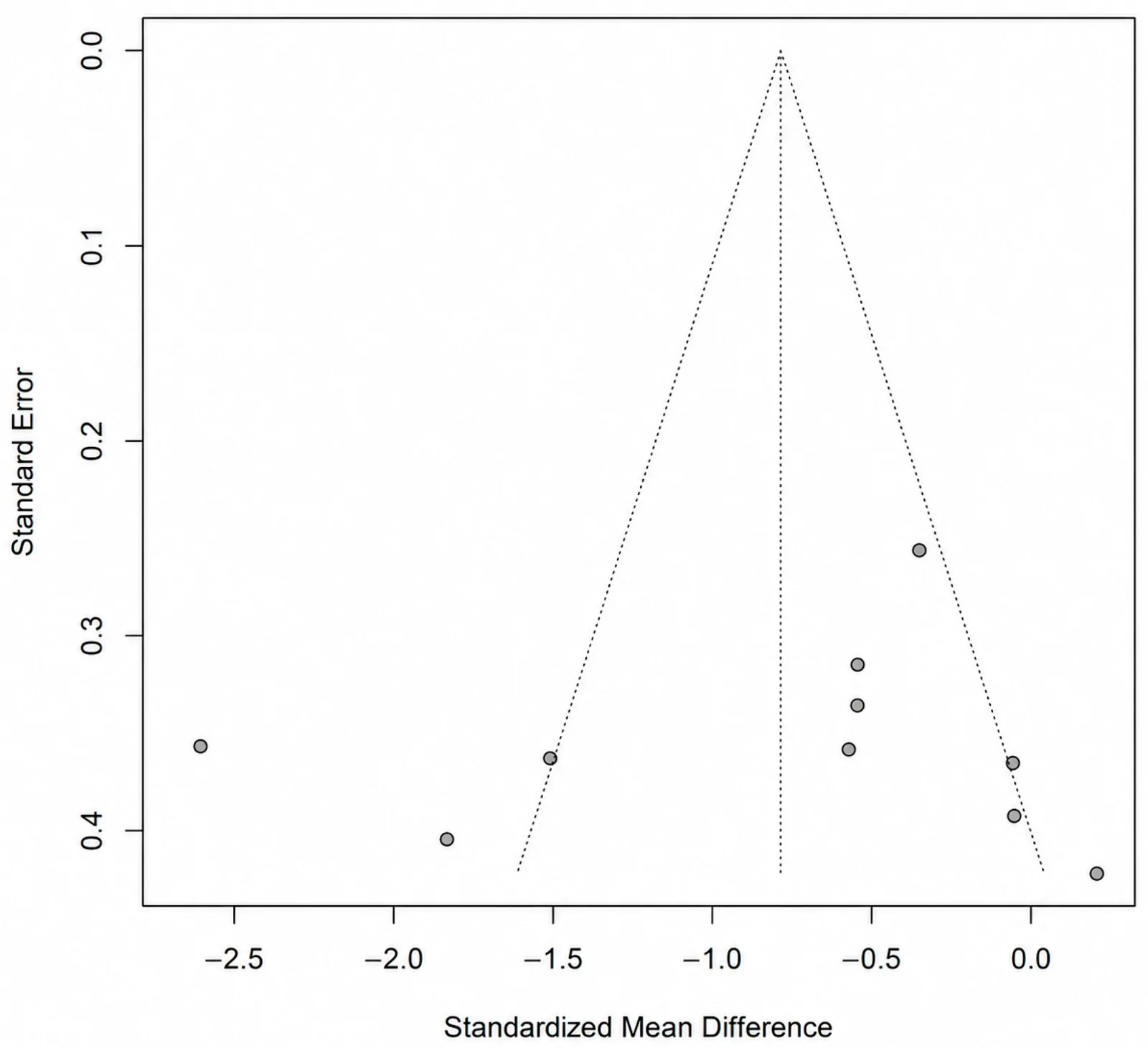
Funnel plot of joint position reproduction error. Each dot represents an individual study. The vertical dotted line indicates the overall pooled effect estimate, and the diagonal dotted lines represent the pseudo 95% confidence limits.

Visual inspection of the funnel plot did not indicate marked asymmetry. Consistently, the Pustejovsky-regression test did not provide statistically significant evidence of funnel-plot asymmetry (test statistic = 1.48, df = 8, *p* = 0.177). However, given the limited number of studies (k = 10) and substantial between-study heterogeneity, this assessment should be interpreted cautiously and does not exclude the possibility of publication or other reporting biases (Figure 6).

### 3.7. GRADE

The certainty of evidence for the primary proprioceptive outcome of joint position reproduction error was rated as very low according to the GRADE assessment. The evidence was downgraded by one level each for risk of bias, inconsistency, and imprecision. Substantial between-study heterogeneity was observed, and the 95% prediction interval crossed the null, supporting the downgrade for inconsistency. No downgrading was applied for indirectness or publication bias, although the assessment of publication bias was limited by the small number of included studies. The certainty of evidence for the subgroup meta-analyses was not separately assessed because of the limited number of studies within each subgroup (Table 6).

## 4. Discussion

### 4.1. Overall Interpretation of Findings

We reviewed 19 randomized studies of interventions addressing lower-limb proprioception after stroke. Ten provided eligible data for the exploratory meta-analysis.

The included studies showed substantial heterogeneity in intervention content, therapeutic rationale, and implementation. Heterogeneity was also evident in proprioceptive assessment, including testing tasks, movement conditions, visual occlusion, measurement devices, and outcome reporting. This methodological heterogeneity limited comparability across studies and complicated interpretation of the pooled results.

These findings informed the development of a mechanism-oriented classification framework that organizes proprioceptive interventions by their therapeutic mechanisms. The present study also provided a systematic summary of the proprioception assessment methods and testing procedures employed across the included studies. The exploratory meta-analysis favored the experimental interventions for JPR angle-error outcomes. However, substantial between-study heterogeneity remained, and the evidence received a certainty rating of very low. Accordingly, the quantitative results remain exploratory and do not establish the superiority of any mechanism category over the others. The proposed framework instead serves to organize heterogeneous interventions and support the development of hypotheses for future research. Further prospective validation of the framework, together with greater standardization of proprioceptive assessment procedures, is needed before stronger mechanistic or clinical conclusions can be drawn.

### 4.2. Peripheral Mechanisms

Based on the mechanism-oriented classification framework, 10 intervention arms were classified as peripheral mechanisms, including interventions involving passive movement, vibration stimulation, Kinesio taping, electrical stimulation, and aquatic exercise. Although these interventions differed in their specific implementation procedures, they shared a common therapeutic characteristic in that they primarily aimed to enhance or modulate afferent sensory input from mechanoreceptors located in muscles, joints, and the skin, thereby altering the availability or characteristics of peripheral sensory information relevant to lower-limb proprioception [2,44,45]. Previous neurophysiological studies have suggested that proprioception depends on multiple sources of sensory input, including muscle spindles, cutaneous mechanoreceptors, and joint receptors, with muscle spindle and cutaneous afferents playing important roles in joint position sense and kinesthesia [46].

Accordingly, interventions targeting peripheral mechanisms may act primarily by modifying receptor stimulation and the resulting somatosensory afferent signals available to the central nervous system. Such modulation may influence the sensory information subsequently used for joint position perception and sensorimotor control. Related evidence indicates that electrical stimulation may enhance motor recovery after stroke [47]. Taping has also been associated with changes in joint proprioception across clinical and healthy populations [48]. These findings provide neurophysiological support for the plausibility of peripheral sensory modulation but do not constitute direct evidence that all interventions classified within this category improve lower-limb proprioception after stroke.

It should be noted that the mechanism classification used in the present study was determined by the intervention component that differed between the experimental and comparator groups rather than by all physiological processes potentially activated during an intervention. Components delivered similarly to both groups were regarded as background or co-interventions and were not used to determine the mechanism category. Similarly, an intervention was not classified as peripheral merely because active movement or exercise inevitably generated proprioceptive afferent input. When the distinguishing therapeutic component primarily emphasized movement-related feedback processing, sensorimotor integration, movement correction, or feedback-based motor control, the intervention was classified according to the corresponding central mechanism criteria. Therefore, classification as a peripheral mechanism indicates that the principal therapeutic target of the between-group intervention contrast was peripheral proprioceptive input; it does not imply that central sensory processing or sensorimotor integration was absent from the resulting response.

### 4.3. Central Mechanisms

Based on the mechanism-oriented classification framework, 8 intervention arms were classified under the central mechanism category. Typical interventions included Tetrax biofeedback balance training, maze control training, visual feedback ankle strategy exercise, repeated active movement, eye-closed treadmill training, task-specific training, and ankle muscle control training using visual feedback. Although these interventions differed in their specific implementation, their distinguishing therapeutic components were primarily directed toward the processing, integration, interpretation, or utilization of sensory and movement-related information for movement correction and motor control.

Visual feedback-based interventions, in particular, may facilitate sensorimotor regulation by providing externally available information about body position, movement performance, or movement error, which can be used to modify subsequent motor output. Other interventions, such as repeated active movement, task-specific training, and eye-closed treadmill training, may place greater demands on the central utilization and integration of movement-related sensory information during active motor control. Motor imagery training was also classified under the central mechanism category because it emphasized internally generated motor representation and cognitive motor control processes in the absence of overt movement. Previous studies have suggested that both mirror visual feedback and motor imagery may serve as neurofacilitation strategies in stroke rehabilitation by promoting activation and reorganization of sensorimotor networks through visual or cognitive representation processes [49]. Evidence from upper-limb stroke rehabilitation has also shown that proprioceptive training incorporating visual feedback may improve proprioceptive and motor outcomes [50].

From a mechanistic perspective, these interventions share an emphasis on how sensory and internally generated movement information is processed and used within the central nervous system, rather than on directly modifying peripheral receptor stimulation. Some of these characteristics overlap with those used in virtual reality-based rehabilitation, including visual feedback, task engagement, and real-time movement-related information [51]. Previous systematic reviews of VR interventions in stroke rehabilitation have also suggested that VR training may improve static postural control and dynamic balance [52]. However, such findings provide only indirect mechanistic context and should not be interpreted as direct evidence for the effectiveness of the central mechanism category defined in the present review. Accordingly, the central mechanism category included interventions whose distinguishing therapeutic component primarily emphasized central sensory processing, sensorimotor integration, movement representation, or feedback-based motor control.

### 4.4. Combined Peripheral and Central Mechanisms

Based on the mechanism-oriented classification framework, three intervention arms were classified under the combined peripheral and central mechanism category. These included cognitive sensorimotor exercise, multi-sensorimotor training combined with transcutaneous electrical nerve stimulation, and robot-guided ankle proprioceptive training combined with thermal-tactile stimulation. Interventions were assigned to this category when the distinguishing therapeutic component incorporated both direct modulation of peripheral proprioceptive input and central sensory processing or sensorimotor integration, and when neither component could reasonably be regarded as incidental or secondary. Thus, the combined category was intended to represent interventions in which peripheral sensory modulation and central processing were both essential components of the therapeutic contrast, rather than interventions that merely contained multiple treatment elements.

From a neurophysiological perspective, recovery of proprioceptive function after stroke may involve a multilevel sensorimotor recovery process [53,54]. Proprioceptive function depends not only on peripheral sensory input but also on complex processes including central sensory integration, motor representation, error correction, and motor control [55]. This multilevel organization provides a theoretical rationale for considering interventions that engage different levels of the proprioceptive and sensorimotor system. After stroke, proprioceptive recovery may therefore involve both the availability of afferent sensory information and the capacity of the central nervous system to interpret and utilize that information for movement control [19,56]. Accordingly, simultaneous engagement of peripheral and central processes represents a plausible therapeutic concept. However, this theoretical rationale does not establish that interventions targeting combined peripheral and central mechanisms are clinically more effective than interventions primarily targeting one level of the sensorimotor system.

In the exploratory subgroup meta-analysis, the subgroup of interventions targeting combined peripheral and central mechanisms showed a favorable within-subgroup pooled effect. However, comparison across the mechanism subgroups did not reveal a statistically significant difference. Therefore, the finding observed within the combined subgroup does not establish the superiority of interventions targeting combined peripheral and central mechanisms over interventions targeting peripheral or central mechanisms alone. Rather, the result should be regarded as a hypothesis-generating observation that supports further investigation of multilevel proprioceptive rehabilitation strategies. With only a limited number of interventions assigned to this category, substantial heterogeneity characterizing the available evidence, and overall certainty rated as very low for the quantitative synthesis, whether interventions simultaneously targeting peripheral and central processes provide additional benefits over approaches primarily targeting a single mechanism remains uncertain and requires direct comparative evaluation in future randomized studies.

### 4.5. Discussion of Proprioception Measurement Methods

Following a systematic review of the assessment methods used in the included studies, the present study found that although JPR was the most frequently used outcome measure for lower-limb proprioception in individuals with stroke, substantial heterogeneity remained in testing procedures and outcome reporting. Even among studies using JPR, considerable variation was observed regarding active versus passive testing conditions, visual occlusion procedures, target angle selection, number of repetitions, and measurement devices. Assessment tools ranged from conventional goniometers and electrogoniometers to isokinetic systems. Furthermore, outcomes were reported in different forms, including angular error, accuracy rate, and number of correct responses. Some studies reported only median and interquartile range (IQR) values or presented data solely in graphical formats, limiting the availability of data for secondary quantitative analyses. As a result, studies employing the same assessment label often remained difficult to compare directly, thereby reducing the feasibility of quantitative evidence synthesis.

In addition to procedural inconsistencies, different proprioception assessment methods may reflect distinct functional constructs. Previous studies have suggested that JPR, TTDPM, and AMEDA are all used to evaluate proprioceptive function, but they do not necessarily assess identical neurophysiological processes [21]. JPR primarily reflects joint position reproduction ability and involves position memory, motor execution, and sensorimotor integration processes. In contrast, TTDPM focuses on the ability to detect the onset of passive joint movement and is generally considered to reflect kinesthetic perception, whereas AMEDA emphasizes the discrimination of movement amplitude and direction [2,21]. Among the 19 studies included in the present review, only one study used TTDPM, whereas the majority relied on JPR as the primary outcome measure. Consequently, current evidence regarding lower-limb proprioceptive recovery after stroke is largely based on JPR outcomes, while the relationships among different dimensions of proprioceptive function remain insufficiently understood.

Most included studies relied on JPR, which primarily evaluates the ability to reproduce or match a target joint position. However, proprioception is multidimensional, and other assessment methods may capture different aspects of proprioceptive function. For example, TTDPM, which was also used in one of the included studies, evaluates the ability to detect the onset of passive joint movement and is commonly used as a measure of kinesthetic perception [21,57]. AMEDA, in contrast, requires participants to discriminate differences in movement extent and may therefore reflect another aspect of proprioceptive function [21]. These methodological differences suggest that JPR, TTDPM, and AMEDA should not be regarded as interchangeable measures of proprioception. Nevertheless, current evidence is insufficient to determine which assessment method is most appropriate for specific intervention mechanisms, clinical contexts, or stages of recovery. Future studies should therefore explore the complementary use of different proprioceptive assessments and directly compare their responsiveness and interpretability across intervention types. Such work may help clarify when individual assessment methods, or combinations of methods, are most informative for evaluating proprioceptive recovery after stroke.

Overall, the major challenge in current lower-limb proprioception research after stroke may not be the lack of assessment tools, but rather the absence of a standardized measurement framework. Inconsistencies in testing procedures, outcome reporting, and the underlying constructs being measured increase the difficulty of interpreting findings and limit the development of robust quantitative evidence. Establishing standardized assessment protocols and clarifying the functional dimensions reflected by different assessment methods may therefore represent an important direction for future research.

### 4.6. Interpretation of Heterogeneity in the Exploratory Meta-Analysis and Quality of Results

The primary purpose of the exploratory meta-analysis conducted in the present study was not to establish definitive conclusions regarding the clinical effectiveness of specific proprioceptive interventions, but rather to explore the available quantitative evidence within the proposed mechanism-oriented classification framework. Ten studies reporting JPR angle-error outcomes were included in the quantitative synthesis. The overall pooled estimate favored the experimental interventions (SMD = −0.78, 95% CI −1.30 to −0.25); however, substantial between-study heterogeneity remained (I^2^ = 82%). Moreover, the 95% prediction interval (−2.42 to 0.87) crossed the null, indicating considerable uncertainty regarding how large the true effect would be, and potentially which direction it would take, in a future study conducted under comparable conditions. Assessment using GRADE yielded a very low certainty rating for the primary outcome. Accordingly, the pooled estimate provides an exploratory overview of the available quantitative evidence but does not permit a definitive conclusion about clinical effectiveness.

Sensitivity analyses provided some evidence that the direction of the pooled estimate was relatively stable, but they did not resolve the substantial heterogeneity. Estimates obtained using the DerSimonian–Laird and restricted maximum likelihood models were generally consistent in direction, and leave-one-out analyses did not materially alter the overall direction of effect. Nevertheless, substantial residual heterogeneity persisted across these analyses. The leave-one-out analysis indicated that Mao et al. (2022) [41] had a relatively large influence on between-study heterogeneity. This appeared to be primarily related to its comparatively large standardized effect estimate. In this study, the post-intervention JPR error was 2.76 ± 0.41° in the experimental group and 3.83 ± 0.40° in the control group, resulting in a relatively large between-group difference in relation to the small within-group variability. Notably, the intervention and proprioceptive outcome assessment were both performed using the same robotic system and involved closely related ankle joint-position tasks. It is therefore possible that task-specific familiarity or device-specific familiarity contributed to the observed study-specific effect estimate. However, this interpretation is speculative, as the original study did not directly examine whether similarity between the training and assessment procedures influenced the measured treatment effect. The available data therefore do not allow the contribution of this factor to be distinguished from the effects of the intervention itself or other methodological characteristics.

Several factors may have contributed to the remaining heterogeneity. First, the included interventions differed substantially in therapeutic content and in the mechanisms they were designed to target. Second, although the quantitative synthesis was restricted to JPR angle-error outcomes, the JPR protocols were not methodologically identical, with differences in active versus passive testing, joint assessed, target angles, visual conditions, numbers of repetitions, and measurement devices. Third, both knee- and ankle-joint proprioceptive outcomes were included, which may represent different biomechanical and sensorimotor contexts. In addition, differences in participant characteristics, intervention duration, and background rehabilitation may also have contributed to between-study variability. The use of SMD allowed outcomes to be expressed on a common statistical scale but did not eliminate these methodological differences or establish equivalence among the underlying JPR protocols.

Taken together, the substantial residual heterogeneity, the prediction interval crossing the null, and an evidence certainty rating of very low call for caution when interpreting the current quantitative findings. The present analysis demonstrates that the proposed mechanism-oriented framework can be applied to organize the available evidence, but it does not establish that the framework improves the interpretability of treatment effects or that any mechanism category is superior to another. Evaluating whether the observed findings are reproducible and clinically relevant will require further randomized studies that use more standardized proprioceptive assessment procedures, specify intervention mechanisms clearly, and recruit larger samples.

### 4.7. Clinical Implications

The mechanism-oriented classification framework proposed in this review may provide clinicians and researchers with a structured way to describe the principal therapeutic targets of lower-limb proprioceptive interventions after stroke. However, the framework should not currently be interpreted as a validated tool for selecting one intervention mechanism over another. Given the substantial heterogeneity of the included studies and the very low certainty of evidence, the available findings are insufficient to support definitive clinical recommendations regarding the relative effectiveness of interventions targeting peripheral mechanisms, central mechanisms, or combined peripheral and central mechanisms.

Although the subgroup of interventions targeting combined peripheral and central mechanisms showed a favorable within-subgroup effect in the exploratory meta-analysis, the formal test for differences between mechanism subgroups was not statistically significant. Therefore, the current evidence does not support the clinical superiority of these interventions over interventions targeting peripheral or central mechanisms alone. The multilevel interaction between peripheral sensory input and central sensorimotor processing remains a plausible theoretical basis for further investigation [19], but whether simultaneously targeting both levels provides additional clinical benefit requires direct comparative evaluation in future randomized studies.

Finally, clinicians and researchers should be aware that proprioceptive outcomes obtained using different assessment protocols may not be directly interchangeable. Even when JPR angle-error outcomes are treated as reflecting a common outcome construct, differences in active versus passive testing, joint assessed, target angle, visual conditions, repetition protocols, and measurement devices may influence the interpretation of the results. Therefore, clinical interpretation of proprioceptive change should consider the specific measurement protocol rather than relying solely on the general label of “JPR.” Greater standardization of proprioceptive assessment and outcome reporting is needed before intervention effects can be compared more confidently across studies and translated into more specific clinical recommendations.

### 4.8. Strengths and Limitations

Rather than focusing solely on intervention effectiveness, this review integrated intervention mechanisms and proprioceptive assessment methodology within a single analytical framework. A mechanism-oriented classification was developed using explicit operational criteria, and the classification process was independently performed by two reviewers, with almost perfect agreement. In addition, the review systematically characterized the substantial variability in proprioceptive assessment procedures, particularly within JPR-based outcomes. The exploratory meta-analysis was supplemented by prediction intervals, model-based sensitivity analyses, leave-one-out analyses, and GRADE assessment, allowing the uncertainty surrounding the pooled findings to be more explicitly characterized. These features provide a structured basis for organizing heterogeneous literature and for generating hypotheses for future mechanism-oriented research.

Several limitations of the underlying evidence should, however, be acknowledged. First, the number of studies available for quantitative synthesis remained limited, and the number of intervention arms within each mechanism subgroup was small. Consequently, subgroup estimates were imprecise and did not provide a sufficient basis for concluding that one mechanism was superior to another. Second, substantial residual heterogeneity persisted despite restricting the quantitative synthesis to JPR angle-error outcomes. Differences remained in intervention content, joint assessed, active versus passive testing, target angles, visual conditions, measurement devices, repetition protocols, participant characteristics, and background rehabilitation. The use of SMD enabled statistical standardization but did not eliminate these methodological differences or establish equivalence among JPR protocols. Third, the meta-analysis was restricted to JPR angle-error outcomes because sufficient quantitative data were not available for other proprioceptive measures. Therefore, the pooled findings may not be generalizable to other dimensions of proprioception assessed using TTDPM, AMEDA, or other measurement approaches. Consistent with these limitations, the certainty of evidence for the primary quantitative outcome was rated as very low.

Several limitations also relate to the review process itself. Study screening and data extraction were primarily conducted by a single reviewer, with additional consultation used when uncertainty arose, which may have increased the possibility of missed records, eligibility errors, extraction errors, or subjective judgments. Although the mechanism classification was subsequently strengthened through explicit decision rules, independent classification by two reviewers, and assessment of inter-rater agreement, the proposed framework was developed within the present review and has not yet undergone external or prospective validation. In addition, only English-language studies were eligible for inclusion, which may have resulted in the omission of relevant evidence published in other languages. These review-process limitations should be distinguished from the methodological limitations of the included studies themselves.

Taken together, these limitations indicate that the current role of the proposed mechanism-oriented framework is to organize evidence and guide the development of hypotheses; its use as a clinical decision-making model has yet to be validated. Future studies should prospectively apply and test the framework, use more standardized proprioceptive assessment procedures, and provide sufficiently detailed outcome reporting to support more robust quantitative comparisons.

## 5. Conclusions

This study systematically reviewed the current evidence on lower-limb proprioception interventions in individuals with stroke and synthesized existing findings from both intervention mechanism and assessment methodology perspectives. The results demonstrated substantial heterogeneity in intervention approaches; however, the primary intervention mechanisms could be categorized into three groups: peripheral mechanisms, central mechanisms, and combined peripheral and central mechanisms. In addition, considerable variability was identified in proprioception assessment methods, testing procedures, and outcome reporting. Different assessment approaches may reflect distinct dimensions of proprioceptive function, limiting direct comparison and quantitative synthesis across studies.

The exploratory meta-analysis conducted using the proposed mechanism-oriented framework and JPR angle-error outcomes suggested an overall favorable effect of proprioceptive interventions. However, substantial between-study heterogeneity remained, and the evidence received a certainty rating of very low. The subgroup findings did not demonstrate statistically significant differences between mechanism categories and therefore provide no basis for concluding that any specific mechanism is superior to the others. Accordingly, the quantitative findings serve to explore potential effects and inform the development of hypotheses, without establishing definitive conclusions about comparative clinical effectiveness.

Overall, the mechanism-oriented classification framework proposed in this study offers a structured approach for organizing heterogeneous lower-limb proprioceptive interventions according to their principal therapeutic mechanisms. Rather than representing a validated analytical or clinical decision-making model, the framework should currently be regarded as a proposed organizational approach that requires further prospective validation. Greater standardization of proprioceptive assessment methods and outcome reporting, together with further research using clearly defined intervention mechanisms, will be important for improving the comparability and interpretability of future evidence.

## Figures and Tables

**Table 1:** Characteristics of the 19 included randomized studies.

Author /Year	Study Design	Sample Size (*n*, M/F)	Age (Years)	Stroke Phase	Intervention Group	Control Group	Intervention Duration and Frequency
Battesha 2022 [33]	2-arm, RCT	Total: 30 Ex1: 15 (10/5) Con2: 15 (11/4)	Ex1: 62.27 ± 1.31 Con2: 61.8 ± 1.12	Chronic stroke	1. Conventional physical therapy 2. The Maze Control Training	1. Conventional physical therapy 2. Selected Proprioceptive Training	Ex1: 60 + 20 min each time/3 days a week/8 weeks Con2: 60 min each time/3 days a week/8 weeks
Cho 2021 [38]	2-arm, RCT	Total:23 Ex1: 13 (10/3) Con2: 10 (8/2)	Ex1: 58.0 ± 6.5 Con 2: 57.2 ± 9.7	Chronic stroke	1. Ankle muscle control training using visual feedback	1. Ankle-related physical therapy 2. Functional electrical stimulation	Ex1: 40 min each time/5 days a week/4 weeks Con2: 40 min each time/5 days a week/4 weeks
Cho 2022 [37]	2-arm, RCT	Total: 30 Ex1: 18 (14/4) Con2: 12 (9/3)	Ex1: 51.8 ± 12.0 Con 2: 55.0 ± 10.9	Chronic stroke	1. Conventional physical therapy 2. Ankle movement training with electrical stimulation therapy	1. Conventional physical therapy 2. Electrical stimulation therapy	Ex1: 90 + 40 min each time/5 days a week/4 weeks Con2: 90 + 40 min each time/5 days a week/4 weeks
Dursun 2025 [35]	2-arm, RCT	Total: 40 Ex1: 20 (10/10) Con2: 20 (10/10)	Ex1: 61.20 ± 9.05 Con2: 65.75 ± 4.63	Chronic stroke	1. Conventional physical therapy 2. Repetitive plantar vibration training	1. Conventional physical therapy 2. Sham vibration	Ex1: 60 + 30 min each day/3 days Con2: 60 + 30 min each day/3 days
Han 2013 [30]	2-arm, RCT	Total: 62 Ex1: 31 (15/16) Con2: 31 (13/18)	Ex1: 56.1 ± 7.3 Con2: 56.6 ± 10.0	Chronic stroke	1. Conventional physical therapy 2. Underwater proprioceptive sense exercise	1. Conventional physical therapy 2. Land unstable proprioceptive sense exercise	Ex1: 40 min each time/3 days a week/6 weeks Con2: 40 min each time/3 days a week/6 weeks
Hung 2016 [25]	2-arm, RCT	Total: 23 Ex1: 12(8/4) Con2: 11(8/3)	Ex1: 52.75 (46.70; 62.90) Con2:55.20 (45.30; 65.50) median (Q1, Q3)	Chronic stroke	1. Conventional physical therapy 2. Tetrax biofeedback balance training	1. Conventional physical therapy	Ex1: 100 + 20 min each time/3 days a week/6 weeks Con2: 100 min each time/3 days a week/6 weeks
Jeon 2019 [29]	2-arm, RCT	Total: 26 Ex1: 13 (7/6) Con2: 13 (7/6)	Ex1: 67.84 ± 11.96 Con2: 67.69 ± 14.45	Chronic stroke	1. Visual feedback group with ankle joint strategy exercise	1. Visual disuse group with ankle joint strategy exercise	Ex1: 30 min each time/3 days a week/6 weeks Con2: 30 min each time/3 days a week/6 weeks
Kim 2021 [26]	3-arm, RCT	Total: 37 Ex1: 13 (7/6) Ex2: 12 (7/5) Con3: 12 (8/4)	Ex1: 50.23 ± 14.89 Ex2: 52.75 ± 17.00 Con3: 55.08 ± 10.55	Chronic stroke	Ex1: 1. Task-specific training 2. Cognitive sensorimotor exercise Ex2: 1. Conventional physical therapy 2. Task-specific training	1. Conventional physical therapy	Ex1: 30 + 30 min each time/5 days a week/8 weeks Ex2: 30 + 30 min each time/5 days a week/8 weeks Con3: 30 min each time/5 days a week/8 weeks
Kim 2022 [27]	2-arm, RCT	Total: 40 Ex1: 20 (11/9) Con2: 20 (13/7)	Ex1: 53.70 ± 9.63 Con2: 54.45 ± 7.28	Chronic stroke	1. Proprioception training with kinesio taping	1. Proprioception training with placebo kinesio taping	Ex1: 30 min each time/5 days a week/4 weeks Con2: 30 min each time/5 days a week/4 weeks
Kwon 2013 [34]	3-arm, RCT	Total: 45 Ex1: 15 (10/5) Ex2: 15 (6/9) Con3: 15 (9/6)	Ex1: 58.67 ± 8.18 Ex2: 61.20 ± 7.98 Con3: 63.33 ± 8.81	Chronic stroke	Ex1: Repeated passive movement Ex2: Repeated active movement	No	Ex1: 15 min × 3 Ex2: 15 min × 3 Con3: 15 min × 3
H Lee 2015 [40]	2-arm, RCT	Total: 36 Ex1: 18 (11/7) Con2: 18 (9/9)	Ex1: ˂65 (*n* = 14), ≥65 (*n* = 4) Con2: ˂65 (*n* = 14), ≥65 (*n* = 4)	Chronic stroke	1. Proprioceptive training accompanied by motor imagery training	1. Proprioceptive training	Ex1: 25 + 5 min each time/5 days a week/8 weeks Con2: 30 min each time/5 days a week/8 weeks
S Lee 2019 [42]	2-arm, RCT	Total: 20 Ex1: 10 (6/4) Con2: 10 (6/4)	Ex1: 69.60 ± 3.98 Con2: 67.70 ± 4.30	Subacute stroke	1. Neurodevelopmental Therapy 2. High frequency Transcutaneous electrical nerve stimulation (TENS)	1. Neurodevelopmental Therapy 2. Low frequency Transcutaneous electrical nerve stimulation (TENS)	Ex1: 30 min each time/5 days a week/4 weeks Con2: 30 min each time/5 days a week/4 weeks
Lim 2019 [28]	2-arm, RCT	Total: 37 Ex1: 19 (11/8) Con2: 18 (10/8)	Ex1: 62.00 ± 7.30 Con2: 59.61 ± 6.77	Subacute stroke	1. Conventional physical therapy 2. Multi-Sensorimotor Training 3. Transcutaneous electrical nerve stimulation	1. Conventional physical therapy 2. Treadmill Gait Training 3. Placebo transcutaneous electrical nerve stimulation	Ex1: 30 + 30 min each time/5 days a week/8 weeks Con2: 30 + 30 min each time/5 days a week/8 weeks
Mao 2022 [41]	2-arm, RCT	Total: 60 Ex1: 30 (20/10) Con2: 30 (18/12)	Ex1: 60.00 ± 9.10 Con2: 61.10 ± 7.88	Chronic stroke	1. Conventional physical therapy 2. Ankle sensory training with robot guidance 3. Thermal-tactile stimulation	1. Conventional physical therapy	Ex1: once a day/5 days a week/6 weeks Con2: once a day/5 days a week/6 weeks
Mazuchi 2018 [31]	2-arm, RCT	Total: 12 Ex1: 6 (1/5) Con2: 6 (1/5)	Ex1: 61.7 ± 10.0 Con2: 54.8 ± 7.7	Chronic stroke	1. Deep-water standing and walking aerobic training	1. Treadmill walking aerobic training	Ex1: 40 min each time/3 days a week/9 weeks Con2: 40 min each time/3 days a week/9 weeks
Moon 2015 [32]	2-arm, RCT	Total: 30 Ex1: 15 (12/3) Con2: 15 (11/4)	Ex1: 55.7 ± 11.7 Con2: 50.1 ± 15.9	Chronic stroke	1. Treadmill training with their eyes closed	1. Treadmill training with their eyes open	Ex1: 30 min each time/3 days a week/4 weeks Con2: 30 min each time/3 days a week/4 weeks
Tikac 2025 [43]	2-arm, RCT	Total: 42 Ex1: 21 Con2: 21	Ex1: 51.71 ± 12.09 Con2: 52.00 ± 10.66	Chronic stroke	1. Bobath exercises 2. Sensory training	1. Bobath exercises	Ex1: 45 min each time/3 days a week/4 weeks Con2: 45 min each time/3 days a week/4 weeks
Tyson 2013 [39]	Randomized crossover trial	Total: 29 (14/15)	Total: 64.5 ± 12.6	Chronic stroke	1. Active transcutaneous electrical nerve stimulation (TENS) applied to the ankle during task performance 2. Sham/control condition (electrodes applied with no electrical stimulation)	No	Single-session intervention (acute effect); crossover design with randomized order of conditions
Xu 2024 [36]	2-arm, RCT	Total: 35 Ex1: 17 (8/9) Con2: 18 (11/7)	Ex1: 58.9 ± 9.74 Con2: 54.17 ± 10.842	Chronic stroke	1. Conventional physical therapy 2. Whole-body vibration (WBV) training	1. Conventional physical therapy	Ex1: 200 + 30 min each time/6 days a week/4 weeks Con2: 200 min each time/6 days a week/4 weeks

Ex: Experimental group, Con: Control group.

**Table 2:** Classification of intervention main mechanisms.

Study/Year	Joint	Intervention Name	Intervention Mechanisms	Combined with Conventional Treatment
Hung 2016 [25]	knee joint	Tetrax Biofeedback balance training	Central mechanisms	Yes
Kim 2021 [26]	knee joint	Experiment I: Cognitive sensorimotor exercise Experiment II: Task-specific training	Experiment I: Combined peripheral and central mechanisms Experiment II: Central mechanisms	No
Kim 2022 [27]	knee joint	Proprioception training with kinesio taping	Peripheral mechanisms	Yes
Lim 2019 [28]	knee joint	Multi-sensorimotor training with transcutaneous electrical nerve stimulation	Combined peripheral and central mechanisms	Yes
Jeon 2019 [29]	knee joint	Visual feedback training ankle joint strategy exercise	Central mechanisms	No
Han 2013 [30]	knee joint	Underwater proprioceptive sense exercise	Peripheral mechanisms	Yes
Moon 2015 [32]	knee joint	Treadmill training with their eyes closed	Central mechanisms	Yes
Battesha 2022 [33]	knee joint	The Maze Control Training	Central mechanisms	Yes
Kwon 2013 [34]	knee joint	Experiment I: repeated passive movement Experiment II: repeated active movement	Experiment I: Peripheral mechanisms Experiment II: Central mechanisms	Not reported
Mazuchi 2018 [31]	knee joint	Deep-water standing and walking aerobic training	Peripheral mechanisms	No
Dursun 2025 [35]	ankle joint	Repetitive plantar vibration	Peripheral mechanisms	Yes
Xu 2024 [36]	ankle joint	Whole-body vibration	Peripheral mechanisms	Yes
Cho 2022 [37]	ankle joint	Passive biaxial ankle movement training	Peripheral mechanisms	Yes
Cho 2021 [38]	ankle joint	Ankle muscle control training using visual feedback	Central mechanisms	No
Tyson 2013 [39]	ankle joint	Transcutaneous electrical nerve stimulation (TENS)	Peripheral mechanisms	No
H Lee 2015 [40]	ankle joint	Motor imagery training	Central mechanisms	Not reported
Mao 2022 [41]	ankle joint	Robot-guided ankle proprioceptive training combined with thermal-tactile stimulation	Combined peripheral and central mechanisms	Yes
S Lee 2019 [42]	ankle joint	High frequency Transcutaneous electrical nerve stimulation (TENS)	Peripheral mechanisms	Yes
Tikac 2025 [43]	ankle joint	Lower-limb sensory training	Peripheral mechanisms	No

**Table 3:** Proprioceptive measures and output units and forms.

Study/Year	Joint	Measurement Methods/Tools	Presentation of Results (Unit)	Summary Format
Hung 2016 [25]	knee joint	JPR/protractor	angle error (°)	medians [first quartiles; third quartiles]
Kim 2021 [26]	knee joint	Active JPR/electrogoniometer; 60 cm × 60 cm × 1 acrylic assessment board	angle error (°)	Mean ± SD
Kim 2022 [27]	knee joint	Active JPR/electrogoniometer; 60 cm × 60 cm × 1 acrylic assessment board	angle error (°)	Mean ± SD
Lim 2019 [28]	knee joint	Active JPR/electrogoniometer; 60 cm × 60 cm × 1 acrylic assessment board	angle error (°)	Mean ± SD
Jeon 2019 [29]	knee joint	Active JPR	angle error (°)	Mean ± SD
Han 2013 [30]	knee joint	Active JPR/Biometrics (Biometrics Ltd. UK)	angle error (°)	Mean ± SD
Moon 2015 [32]	knee joint	Passive JPR/Isokinetic equipment (Biodex System Pro 3‚ Bio dex Medical System, Inc.‚ NY‚ USA)	angle error (°)	Mean ± SD
Battesha 2022 [33]	knee joint	Passive JPR/Biodex System 3 Isokinetic Dynamometer	Not reported	Mean ± SD
Kwon 2013 [34]	knee joint	Active JPR; passive JPR/Biodex (Biodex Multi-Joint System 2, Biodex Corp, NY, USA)	(°) %	Mean ± SD
Mazuchi 2018 [31]	knee joint	Passive JPR/isokinetic dynamometer (Biodex, System 3, USA)	absolute error (AE, °) variable error (VE, °)	figure
Dursun 2025 [35]	ankle joint	Passive JPR/HUMAC NORM^®^ Isokinetic Extremity System (Computer Sports Medicine Inc., Stoughton, MA, USA)	angle error (°)	IQR
Xu 2024 [36]	ankle joint	Active JPR/TecnoBody PROKIN System (PK252)	Number of hits per target (number of times) success rate (%)	figure/mean/Pearson correlation coefficient
Cho 2022 [37]	ankle joint	Passive JPR	success rate (%)	Mean ± SD
Cho 2021 [38]	ankle joint	Passive JPR	angle error (°)	Mean ± SD
Tyson 2013 [39]	ankle joint	TTDPM/Biodex^®^ Isokinetic Dyanamoeter	Minimum motion perception angle (°)	Mean ± SD
H Lee 2015 [40]	ankle joint	JPR	angle error (°)	Mean ± SD
Mao 2022 [41]	ankle joint	Active JPR/AnkleMotus M1A produced by Shanghai Fourier Intelligent Technology Co., Ltd.	angle error (°)	Mean ± SD
S Lee 2019 [42]	ankle joint	Passive JPR/Biodex Multi-joint System 3; Biodex Medical System Inc., New York, NY, USA	angle error (°)	Mean ± SD
Tikac 2025 [43]	ankle joint	Active JPR/HALO brand digital goniometer	angle error (°)	Mean ± SD

**Table 4:** Comparison of random-effects models using DL and REML estimators.

Model	SMD	SE	95% CI	*p* Value	τ^2^	I^2^ (%)	95% Prediction Interval
DL	−0.78	0.27	−1.30 to −0.25	0.004	0.63	82.38	−2.42 to 0.87
REML	−0.78	0.27	−1.31 to −0.24	0.004	0.66	83.06	−2.46 to 0.91

DL, DerSimonian–Laird; REML, restricted maximum likelihood; SMD, standardized mean difference; SE, standard error; CI, confidence interval.

**Table 5:** Leave-one-out sensitivity analysis of the overall pooled effect.

Omitted Study	SMD	95% CI	*p* Value	τ^2^	I^2^ (%)
Cho 2021 [38]	−0.87	−1.41 to −0.33	0.002	0.62	82.41
H Lee 2015 [40]	−0.67	−1.21 to −0.14	0.014	0.59	81.67
Han 2013 [30]	−0.82	−1.41 to −0.24	0.006	0.74	83.24
Jeon 2019 [29]	−0.85	−1.41 to −0.29	0.003	0.66	83.10
Kim 2021 [26]	−0.81	−1.40 to −0.22	0.007	0.69	85.10
Kim 2022 [27]	−0.70	−1.26 to −0.15	0.013	0.65	82.70
Lim 2019 [28]	−0.80	−1.38 to −0.22	0.007	0.73	84.02
Mao 2022 [41]	−0.58	−0.99 to −0.18	0.005	0.27	66.71
Moon 2015 [32]	−0.85	−1.41 to −0.29	0.003	0.66	82.93
Tikac 2025 [43]	−0.80	−1.39 to −0.22	0.007	0.74	84.00

The direction of effect remained unchanged and all confidence intervals excluded zero after omission of each individual study. Abbreviations: SMD, standardized mean difference; CI, confidence interval.

**Table 6:** GRADE assessment of the certainty of evidence for proprioceptive outcomes in patients with stroke.

Outcome	RoB 2	Inconsistency	Indirectness	Imprecision	Publication Bias	Certainty
Primary outcome	Serious (−1). Six of the ten studies, accounting for approximately 60% of the pooled weight, were rated as having some concerns in the RoB 2 assessment. These limitations were considered sufficient to reduce confidence in the pooled estimate.	Serious (−1). Substantial unexplained heterogeneity was observed (I^2^ = 82%), with marked variation in the magnitude of individual study effects. The 95% prediction interval crossed the null (−2.42 to 0.87), and substantial heterogeneity persisted in subgroup and leave-one-out analyses.	Not serious (0). All included studies directly addressed the prespecified population, intervention, comparator, and proprioceptive outcome.	Serious (−1). The pooled 95% CI did not cross the null (SMD = −0.78, 95% CI −1.30 to −0.25); however, the total sample size (*n* = 393) did not meet the GRADE rule-of-thumb optimal information size of 800 participants for continuous outcomes.	Not serious (0). Visual inspection of the funnel plot did not indicate marked asymmetry, and the Pustejovsky-Rodgers regression test showed no statistically significant evidence of funnel-plot asymmetry (*p* = 0.177). No downgrade was applied for publication bias; however, this assessment was limited by the small number of studies (k = 10) and substantial between-study heterogeneity.	Very low

GRADE, Grading of Recommendations Assessment, Development and Evaluation; RoB 2, Risk of Bias 2; SMD, standardized mean difference; CI, confidence interval; OIS, optimal information size. −1, downgraded by one level; 0, no downgrading.

## Data Availability

The data supporting the findings of this study are available within the article and its Supplementary Materials. No new primary data were generated in this study.

## Supplementary Materials

The following supporting information can be downloaded at https://www.mdpi.com/article/10.3390/jcm15197629/s1.

## Abbreviations

The following abbreviations are used in this manuscript: AE, absolute error; AMEDA, active movement extent discrimination assessment; CI, confidence interval; CT, computed tomography; CVA, cerebrovascular accident; DL, DerSimonian–Laird; GRADE, Grading of Recommendations Assessment, Development and Evaluation; IQR, interquartile range; JPR, joint position reproduction; MRI, magnetic resonance imaging; PRISMA, Preferred Reporting Items for Systematic Reviews and Meta-Analyses; PROSPERO, International Prospective Register of Systematic Reviews; RCT, randomized controlled trial; REML, restricted maximum likelihood; RoB 2, Cochrane risk-of-bias tool for randomized trials, version 2; SD, standard deviation; SE, standard error; SMD, standardized mean difference; TENS, transcutaneous electrical nerve stimulation; TTDPM, threshold to detection of passive motion; VE, variable error; VR, virtual reality; WBV, whole-body vibration.
