# Supplementary material for: An Integrated Framework for Classifying Intervention Mechanisms and Measurement Methodologies of Lower-Limb Proprioception in Patients with Stroke: A Systematic Review and Exploratory Meta-Analysis
Source: J Clin Med. 2026 Oct 1;15(19):7629. doi: 10.3390/jcm15197629 (PMC13666519; doi:10.3390/jcm15197629)
Supplement: Supplementary file 1 [file jcm-15-07629-s001.zip › Detailed platform specific search/search history.pdf]

Pub med		
Stroke	#1: "stroke"[Title/Abstract] OR "apoplexy"[Title/Abstract] OR "CVA"[Title/Abstract] OR "cerebral stroke"[Title/Abstract] OR "cerebrovascular accident acute"[Title/Abstract] OR "cerebrovascular apoplexy"[Title/Abstract] OR "cerebrovascular stroke"[Title/Abstract] OR "stroke acute"[Title/Abstract] OR "vascular accident brain"[Title/Abstract] OR "stroke rehabilitation"[Title/Abstract] OR "cerebrovascular accident"[Title/Abstract]	374,893 results 2025.11.10
proprioception	#2: "proprioception"[Title/Abstract] OR "labyrinthine sense"[Title/Abstract] OR "position sense"[Title/Abstract] OR "sense of equilibrium"[Title/Abstract] OR "sense of position"[Title/Abstract] OR "vestibular sense"[Title/Abstract]	8,882 results 2025.11.10
Stroke+proprioception (Clinical Trial, Randomized Controlled Trial)	#1 AND #2:(("stroke"[Title/Abstract] OR "apoplexy"[Title/Abstract] OR "CVA"[Title/Abstract] OR "cerebral stroke"[Title/Abstract] OR "cerebrovascular accident acute"[Title/Abstract] OR "cerebrovascular apoplexy"[Title/Abstract] OR "cerebrovascular stroke"[Title/Abstract] OR "stroke acute"[Title/Abstract] OR "vascular accident brain"[Title/Abstract] OR "stroke rehabilitation"[Title/Abstract] OR "cerebrovascular accident"[Title/Abstract]) AND ("proprioception"[Title/Abstract] OR "labyrinthine sense"[Title/Abstract] OR "position sense"[Title/Abstract] OR "sense of equilibrium"[Title/Abstract] OR "sense of position"[Title/Abstract] OR "vestibular sense"[Title/Abstract])) AND (clinicaltrial[Filter] OR randomizedcontrolledtrial[Filter])	62 results 2025.11.10 67 results 2026.3.31

Embase		
Stroke	#1:stroke:ti,ab,kw OR apoplexy:ti,ab,kw OR cva:ti,ab,kw OR 'cerebral stroke':ti,ab,kw OR 'cerebrovascular accident,acute':ti,ab,kw OR 'cerebrovascular apoplexy':ti,ab,kw OR 'cerebrovascular stroke':ti,ab,kw OR stroke,acute:ti,ab,kw OR 'vascular accident,brain':ti,ab,kw OR 'stroke rehabilitation':ti,ab,kw OR 'cerebrovascular accident':ti,ab,kw	618,329 results<br>2025,11.10
proprioception	#2: 'proprioception':ti,ab,kw OR 'labyrinthine sense':ti,ab,kw OR 'position sense':ti,ab,kw OR 'sense of equilibrium':ti,ab,kw OR 'sense of position':ti,ab,kw OR 'vestibular sense':ti,ab,kw	13,368 results<br>2025.11.10
Stroke+proprioception (randomized controlled)	#1AND #2 AND 'randomized controlled trial'/de	132 results<br>2025.11.10<br>140 results<br>2026.03.31

Scopus		
Stroke	(TITLE-ABS-KEY (storke) OR TITLE-ABS-KEY (apoplexy) OR TITLE-ABS-KEY (cva) OR TITLE-ABS-KEY (cerebral stroke) OR TITLE-ABS-KEY (cerebrovascular accident , acute) OR TITLE-ABS-KEY (cerebrovascular apoplexy) OR TITLE-ABS-KEY (cerebrovascular stroke) OR TITLE-ABS-KEY (stroke , acute) OR TITLE-ABS-KEY (vascular accident , brain) OR TITLE-ABS-KEY (stroke rehabilitation) OR TITLE-ABS-KEY (cerebrovascular accident))	432,208results 2025.11.11
proprioception	(TITLE-ABS-KEY (proprioception) OR TITLE-ABS-KEY (labyrinthine sense) OR TITLE-ABS-KEY (sense of equilibrium) OR TITLE-ABS-KEY (sense of position) OR TITLE-ABS-KEY (vestibular sense) OR TITLE-ABS-KEY (position sense))	64,734results 2025.11.11
Stroke AND Proprioception (Article,English)	((TITLE-ABS-KEY (proprioception) OR TITLE-ABS-KEY (labyrinthine sense) OR TITLE-ABS-KEY (sense of equilibrium) OR TITLE-ABS-KEY (sense of position) OR TITLE-ABS-KEY (vestibular sense) OR TITLE-ABS-KEY (position sense))) AND ((TITLE-ABS-KEY (storke) OR TITLE-ABS-KEY (apoplexy) OR TITLE-ABS-KEY (cva) OR TITLE-ABS-KEY (cerebral stroke) OR TITLE-ABS-KEY (cerebrovascular accident , acute) OR TITLE-ABS-KEY (cerebrovascular apoplexy) OR TITLE-ABS-KEY (cerebrovascular stroke) OR TITLE-ABS-KEY (stroke , acute) OR TITLE-ABS-KEY (vascular accident , brain) OR TITLE-ABS-KEY (stroke rehabilitation) OR TITLE-ABS-KEY (cerebrovascular accident))) AND (LIMIT-TO (DOCTYPE , "ar")) AND (LIMIT-TO (LANGUAGE , "English"))	836results 2025.11.11 1085 results 2026.03.31

Web of science		
STROKE	#1:stroke (Topic) OR apoplexy (Topic) OR CVA (Topic) OR cerebral stroke (Topic) OR cerebrovascular accident,acute (Topic) OR cerebrovascular apoplexy (Topic) OR cerebrovascular stroke (Topic) OR stroke,acute (Topic) OR vascular accident,brain (Topic) OR stroke rehabilitation (Topic) OR cerebrovascular accident (Topic)	65,255RESULTS 2025.11.10
PROPRIOCEPTION	#2:proprioception (Topic) OR labyrinthine sense (Topic) OR position sense (Topic) OR sense of equilibrium (Topic) OR sense of position (Topic) OR vestibular sense (Topic)	17,705RESULTS 2025.11.10
STROKE AND PROPRIONCEPTION	#2 AND #3	193 RESULTS 2025.11.10 195 RESULTS 2026.03.31

COCHRANE LINRARY		
STROKE	#1:stroke:ti,ab,kw OR apoplexy:ti,ab,kw OR cva:ti,ab,kw OR 'cerebral stroke':ti,ab,kw OR 'cerebrovascular accident,acute':ti,ab,kw OR 'cerebrovascular apoplexy':ti,ab,kw OR 'cerebrovascular stroke':ti,ab,kw OR stroke,acute:ti,ab,kw OR 'vascular accident,brain':ti,ab,kw OR 'stroke rehabilitation':ti,ab,kw OR 'cerebrovascular accident':ti,ab,kw	83,150RESULTS 2025.11.11
PROPRIOCEPTION	#2:'proprioception':ti,ab,kw OR 'labyrinthine sense':ti,ab,kw OR 'position sense':ti,ab,kw OR 'sense of equilibrium':ti,ab,kw OR 'sense of position':ti,ab,kw OR 'vestibular sense':ti,ab,kw	3,944RESULTS 2025.11.11
STROKE AND PROPRIOCEPTION English AND trials	#1 AND #2 AND English AND trials	341 RESULTS 2025.11.11

OVID		
STROKE	#1:(stroke or apoplexy or CVA or cerebral stroke or cerebrovascular accident, acute or cerebrovascular apoplexy or cerebrovascular stroke or stroke, acute or vascular accident, brain or stroke rehabilitation or cerebrovascular accident).mp. [mp=title, book title, abstract, original title, name of substance word, subject heading word, floating sub-heading word, keyword heading word, organism supplementary concept word, protocol supplementary concept word, rare disease supplementary concept word, unique identifier, synonyms, population supplementary concept word, anatomy supplementary concept word]	376,275RESULTS 2025.11.10
PROPRIOCEPTION	#2:(proprioception or labyrinthine sense or position sense or sense of equilibrium or sense of position or vestibular sense).mp. [mp=title, book title, abstract, original title, name of substance word, subject heading word, floating sub-heading word, keyword heading word, organism supplementary concept word, protocol supplementary concept word, rare disease supplementary concept word, unique identifier, synonyms, population supplementary concept word, anatomy supplementary concept word]	12,793RESULTS 2025.11.10
STROKE AND PROPRIOCEPTION (AND humans AND randomized controlled trial)	#1 AND #2 AND humans AND randomized controlled trial	66 RESULTS 2025.11.10
