# Supplementary material for: An Integrated Framework for Classifying Intervention Mechanisms and Measurement Methodologies of Lower-Limb Proprioception in Patients with Stroke: A Systematic Review and Exploratory Meta-Analysis
Source: J Clin Med. 2026 Oct 1;15(19):7629. doi: 10.3390/jcm15197629 (PMC13666519; doi:10.3390/jcm15197629)
Supplement: Supplementary file 1 [file jcm-15-07629-s001.zip › jcm-4549623-Table S1.pdf]

Supplementary Table S1

Complete Database-Specific Search Strategies

Search concepts were adapted to the syntax and available fields of each database. Database-specific Boolean operators, field restrictions, filters, and limits are reported below. The review used a common evidence-coverage cutoff of March 31, 2026. PubMed, Embase, Scopus, and Web of Science were updated on March 31, 2026. To document equivalent coverage for the two databases that were not included in that update, Cochrane Library and Ovid MEDLINE were re-run on September 14, 2026. For Cochrane Library, the date cutoff was applied directly in the interface. For Ovid MEDLINE, the September 14, 2026 search was exported first and records dated after March 31, 2026 were removed during post-export date screening.

Search-history summary

Database	Initial search	Common cutoff / update	Later verification
PubMed	Nov 10, 2025: 62	Mar 31, 2026: 67	
Embase	Nov 10, 2025: 132	Mar 31, 2026: 140	
Scopus	Nov 11, 2025: 836	Mar 31, 2026: 1,085	
Web of Science	Nov 10, 2025: 193	Mar 31, 2026: 195	
Cochrane Library	Nov 11, 2025: 341	Coverage reconstructed through Mar 31, 2026 (366)	Sep 14, 2026 re-run with direct date cutoff: 366
Ovid MEDLINE	Nov 10, 2025: 66	Coverage reconstructed through Mar 31, 2026 (75)	Sep 14, 2026 re-run: 77 before post-export date screening

S1. PubMed

**Database**	PubMed
**Platform**	National Library of Medicine (PubMed)
**Initial search**	November 10, 2025（**Records：** 62）
**Final coverage cutoff**	March 31, 2026（**Records：** 67）
**Search fields**	Title/Abstract
**Interface filters**	Article type: Clinical Trial; Randomized Controlled Trial (applied after combining #1 and #2)
**Records at cutoff**	67

Search strategy

#1 Stroke

```
"stroke"[Title/Abstract] OR "apoplexy"[Title/Abstract] OR "CVA"[Title/Abstract] OR "cerebral
stroke"[Title/Abstract] OR "cerebrovascular accident acute"[Title/Abstract] OR "cerebrovascular
apoplexy"[Title/Abstract] OR "cerebrovascular stroke"[Title/Abstract] OR "stroke
acute"[Title/Abstract] OR "vascular accident brain"[Title/Abstract] OR "stroke
rehabilitation"[Title/Abstract] OR "cerebrovascular accident"[Title/Abstract]
```

#2 Proprioception

```
"proprioception"[Title/Abstract] OR "labyrinthine sense"[Title/Abstract] OR "position
sense"[Title/Abstract] OR "sense of equilibrium"[Title/Abstract] OR "sense of
position"[Title/Abstract] OR "vestibular sense"[Title/Abstract]
```

#3 Combined search before interface filters

```
#1 AND #2
("stroke"[Title/Abstract] OR "apoplexy"[Title/Abstract] OR "CVA"[Title/Abstract] OR "cerebral
stroke"[Title/Abstract] OR "cerebrovascular accident acute"[Title/Abstract] OR "cerebrovascular
```

```
apoplexy"[Title/Abstract] OR "cerebrovascular stroke"[Title/Abstract] OR "stroke
acute"[Title/Abstract] OR "vascular accident brain"[Title/Abstract] OR "stroke
rehabilitation"[Title/Abstract] OR "cerebrovascular accident"[Title/Abstract]) AND
("proprioception"[Title/Abstract] OR "labyrinthine sense"[Title/Abstract] OR "position
sense"[Title/Abstract] OR "sense of equilibrium"[Title/Abstract] OR "sense of
position"[Title/Abstract] OR "vestibular sense"[Title/Abstract]) AND (clinicaltrial[Filter] OR
randomizedcontrolledtrial[Filter])
```

S2. Embase

**Database**	Embase
**Platform**	Embase.com
**Initial search**	November 10, 2025（**Records： 132**）
**Final coverage cutoff**	March 31, 2026（**Records： 140**）
**Search fields**	Title, abstract, and keywords (:ti,ab,kw)
**Study-design restriction**	randomized controlled trial indexed term (/de)<br>(applied after combining #1 and #2)
**Records at cutoff**	140

Search strategy

#1 Stroke

```
stroke:ti,ab,kw OR apoplexy:ti,ab,kw OR cva:ti,ab,kw OR 'cerebral stroke':ti,ab,kw OR
'cerebrovascular accident,acute':ti,ab,kw OR 'cerebrovascular apoplexy':ti,ab,kw OR 'cerebrovascular
stroke':ti,ab,kw OR stroke,acute:ti,ab,kw OR 'vascular accident,brain':ti,ab,kw OR 'stroke
rehabilitation':ti,ab,kw OR 'cerebrovascular accident':ti,ab,kw
```

#2 Proprioception

```
'proprioception':ti,ab,kw OR 'labyrinthine sense':ti,ab,kw OR 'position sense':ti,ab,kw OR 'sense of
equilibrium':ti,ab,kw OR 'sense of position':ti,ab,kw OR 'vestibular sense':ti,ab,kw
```

#3 Combined search

```
#1 AND #2 AND 'randomized controlled trial'/de
```

S3. Scopus

**Database**	Scopus
**Platform**	Elsevier
**Initial search**	November 11, 2025（**Records： 836**）
**Final coverage cutoff**	March 31, 2026（**Records： 1085**）
**Search fields**	Title, abstract, and keywords (TITLE-ABS-KEY)
**Filters/limits**	Document type = Article; Language = English<br>(applied after combining #1 and #2)
**Records at cutoff**	1,085

Search strategy

#1 Stroke

```
TITLE-ABS-KEY(stroke) OR TITLE-ABS-KEY(apoplexy) OR TITLE-ABS-KEY(cva) OR TITLE-ABS-KEY(cerebral
stroke) OR TITLE-ABS-KEY(cerebrovascular accident, acute) OR TITLE-ABS-KEY(cerebrovascular apoplexy)
OR TITLE-ABS-KEY(cerebrovascular stroke) OR TITLE-ABS-KEY(stroke, acute) OR TITLE-ABS-KEY(vascular
accident, brain) OR TITLE-ABS-KEY(stroke rehabilitation) OR TITLE-ABS-KEY(cerebrovascular accident)
```

#2 Proprioception

```
TITLE-ABS-KEY(proprioception) OR TITLE-ABS-KEY(labyrinthine sense) OR TITLE-ABS-KEY(sense of
equilibrium) OR TITLE-ABS-KEY(sense of position) OR TITLE-ABS-KEY(vestibular sense) OR TITLE-ABS-
KEY(position sense)
```

#3 Combined search

```
((TITLE-ABS-KEY(proprioception) OR TITLE-ABS-KEY(labyrinthine sense) OR TITLE-ABS-KEY(sense of
equilibrium) OR TITLE-ABS-KEY(sense of position) OR TITLE-ABS-KEY(vestibular sense) OR TITLE-ABS-
KEY(position sense))) AND ((TITLE-ABS-KEY(stroke) OR TITLE-ABS-KEY(apoplexy) OR TITLE-ABS-KEY(cva)
OR TITLE-ABS-KEY(cerebral stroke) OR TITLE-ABS-KEY(cerebrovascular accident, acute) OR TITLE-ABS-
KEY(cerebrovascular apoplexy) OR TITLE-ABS-KEY(cerebrovascular stroke) OR TITLE-ABS-KEY(stroke,
acute) OR TITLE-ABS-KEY(vascular accident, brain) OR TITLE-ABS-KEY(stroke rehabilitation) OR TITLE-
ABS-KEY(cerebrovascular accident))) AND LIMIT-TO(DOCTYPE, "ar") AND LIMIT-TO(LANGUAGE, "English")
```

Correction of historical typo: The earlier supplementary history contained “storke”. The term was verified and corrected to “stroke” in the reconstructed/updated strategy shown here.

S4. Web of Science

**Database**	Web of Science
**Platform**	Web of Science Core Collection
**Initial search**	November 10, 2025（**Records： 193**）
**Final coverage cutoff**	March 31, 2026（**Records： 195**）
**Search field**	Topic
**Archived index/entitlement codes**	WOS.ISTP (1990–2025); WOS.ISSHP (1990–2025)
**Language / document-type filters**	None documented
**Records at cutoff**	195

Search strategy

#1 Stroke

```
stroke (Topic) OR apoplexy (Topic) OR CVA (Topic) OR cerebral stroke (Topic) OR cerebrovascular
accident, acute (Topic) OR cerebrovascular apoplexy (Topic) OR cerebrovascular stroke (Topic) OR
stroke, acute (Topic) OR vascular accident, brain (Topic) OR stroke rehabilitation (Topic) OR
cerebrovascular accident (Topic)
```

#2 Proprioception

```
proprioception (Topic) OR labyrinthine sense (Topic) OR position sense (Topic) OR sense of
equilibrium (Topic) OR sense of position (Topic) OR vestibular sense (Topic)
```

#3 Combined search

```
#1 AND #2
```

S5. Cochrane Library

**Database**	Cochrane Library / CENTRAL records
**Platform**	Cochrane Library
**Initial search**	November 11, 2025（**Records： 341**）
**Re-run date**	September 14, 2026（**Records： 366**）
**Final coverage cutoff**	March 31, 2026 (applied directly as a custom date range in the interface)
**Search fields**	Title, abstract, and keyword (:ti,ab,kw)
**Interface filters**	Language = English; Content type = Trials; Date = custom range through March 31, 2026 (applied after combining #1 and #2)
**Records at cutoff**	366

Search strategy

#1 Stroke

```
stroke:ti,ab,kw OR apoplexy:ti,ab,kw OR cva:ti,ab,kw OR 'cerebral stroke':ti,ab,kw OR
'cerebrovascular accident,acute':ti,ab,kw OR 'cerebrovascular apoplexy':ti,ab,kw OR 'cerebrovascular
stroke':ti,ab,kw OR stroke,acute:ti,ab,kw OR 'vascular accident,brain':ti,ab,kw OR 'stroke
rehabilitation':ti,ab,kw OR 'cerebrovascular accident':ti,ab,kw
```

#2 Proprioception

```
'proprioception':ti,ab,kw OR 'labyrinthine sense':ti,ab,kw OR 'position sense':ti,ab,kw OR 'sense of
equilibrium':ti,ab,kw OR 'sense of position':ti,ab,kw OR 'vestibular sense':ti,ab,kw
```

#3 Combined search before interface filters

```
#1 AND #2
```

S6. Ovid MEDLINE

**Database**	Ovid MEDLINE(R)
**Platform**	Ovid
**Initial search**	November 10, 2025（**Records： 66**）
**Re-run date**	September 14, 2026（**Records： 77**）
**Final coverage cutoff**	March 31, 2026 (implemented by post-export date screening)
**Search field**	Multipurpose field (.mp.)
**Population limit**	Humans
**Study-design limit**	Randomized controlled trial
**Records retrieved on Sep 14**	77 before post-export date screening<br>After manually deleting records after 3.31（**Records： 75**）

Search strategy

#1 Stroke

```
(stroke or apoplexy or CVA or cerebral stroke or cerebrovascular accident, acute or cerebrovascular
apoplexy or cerebrovascular stroke or stroke, acute or vascular accident, brain or stroke
rehabilitation or cerebrovascular accident).mp.
```

The archived Ovid record defined .mp. as: title, book title, abstract, original title, name of substance word, subject heading word, floating sub-heading word, keyword heading word, organism supplementary concept word, protocol supplementary concept word, rare disease supplementary concept word, unique identifier, synonyms, population supplementary concept word, and anatomy supplementary concept word.

#2 Proprioception

```
(proprioception or labyrinthine sense or position sense or sense of equilibrium or sense of position
or vestibular sense).mp.
```

#3 Combined search and limits

```
#1 AND #2
#3 AND humans
#4 AND randomized controlled trial
```

Date handling: The Ovid search was re-run on September 14, 2026 without an internal March 31 date cutoff. The 77 retrieved records were exported, and records dated after March 31, 2026 were then removed during post-export date screening so that the evidence coverage matched the common review cutoff.
